# Evaluation of a clinical decision support system for rare diseases: a qualitative study

**DOI:** 10.1186/s12911-021-01435-8

**Published:** 2021-02-18

**Authors:** Jannik Schaaf, Martin Sedlmayr, Brita Sedlmayr, Hans-Ulrich Prokosch, Holger Storf

**Affiliations:** 1 Medical Informatics Group (MIG), University Hospital Frankfurt, Frankfurt, Germany; 2 Institute for Medical Informatics and Biometry, Carl Gustav Carus Faculty of Medicine, Technical University of Dresden, Dresden, Germany; 3 Department of Medical Informatics, Biometrics and Epidemiology, Friedrich-Alexander University Erlangen-Nürnberg, Erlangen, Germany

**Keywords:** Rare diseases, Clinical decision support systems, Computer-assisted diagnosis, Usability

## Abstract

**Background:**

Rare Diseases (RDs) are difficult to diagnose. Clinical Decision Support Systems (CDSS) could support the diagnosis for RDs. The Medical Informatics in Research and Medicine (MIRACUM) consortium developed a CDSS for RDs based on distributed clinical data from eight German university hospitals. To support the diagnosis for difficult patient cases, the CDSS uses data from the different hospitals to perform a patient similarity analysis to obtain an indication of a diagnosis. To optimize our CDSS, we conducted a qualitative study to investigate usability and functionality of our designed CDSS.

**Methods:**

We performed a Thinking Aloud Test (TA-Test) with RDs experts working in Rare Diseases Centers (RDCs) at MIRACUM locations which are specialized in diagnosis and treatment of RDs. An instruction sheet with tasks was prepared that the participants should perform with the CDSS during the study. The TA-Test was recorded on audio and video, whereas the resulting transcripts were analysed with a qualitative content analysis, as a ruled-guided fixed procedure to analyse text-based data. Furthermore, a questionnaire was handed out at the end of the study including the System Usability Scale (SUS).

**Results:**

A total of eight experts from eight MIRACUM locations with an established RDC were included in the study. Results indicate that more detailed information about patients, such as descriptive attributes or findings, can help the system perform better. The system was rated positively in terms of functionality, such as functions that enable the user to obtain an overview of similar patients or medical history of a patient. However, there is a lack of transparency in the results of the CDSS patient similarity analysis. The study participants often stated that the system should present the user with an overview of exact symptoms, diagnosis, and other characteristics that define two patients as similar. In the usability section, the CDSS received a score of 73.21 points, which is ranked as good usability.

**Conclusions:**

This qualitative study investigated the usability and functionality of a CDSS of RDs. Despite positive feedback about functionality of system, the CDSS still requires some revisions and improvement in transparency of the patient similarity analysis.

## Background

According to World Health Organization (WHO), a disease is referred to as “rare”, if less than 1.3 out of 2000 people are affected [1]. It is estimated that currently about 7000 different rare diseases (RDs) exist, with about 400 million people affected worldwide [2]. Many of these diseases are chronic, degenerative or life threatening. Additionally, they can lead to life impairment or severe disabilities [3, 4]. While about 80% of the RDs have a genetic origin, infectious, immunological, or environmental factors are also observed as possible disease causes [5–7].

Often, many years pass by until an RD is diagnosed, which lead to late diagnosis or remaining undiagnosed, especially those with phenotypes that occur later in life [3]. The search for the right diagnosis can lead to years of limitations and major suffering for patients [8]. A further problem is the geographical distribution of experts for RDs. Additionally, research and care are restricted due to the existence of only a few studies and limited patient data [9]. Therefore, research networks like the MIRACUM consortium (Medical Informatics in Research and Care in University Medicine), which makes clinical knowledge from various hospitals available, are a promising approach for RDs, in order to generate new knowledge for research and care [10]. One use-case of MIRACUM is the development of a Clinical Decision Support System (CDSS) for RDs, which is called DISERDIS (Diagnosis Support in Rare Diseases). The aim of DISERDIS is to identify similar patients at other MIRACUM locations, which could give an indication for a diagnosis [11].

During the development of a CDSS, it is important to involve future users in every phase of development to ensure acceptance and purposefulness of the system in everyday clinical practice [12–16]. Therefore, we decided to use a “User-Centred Design Process” (UCD) for the development of DISERDIS. In the context of the UCD, we took several steps to identify user-requirements and needs, including a scoping review of literature about CDSS for RDs [17], a cross sectional survey [18], a qualitative study with expert interviews [19] and a focus group. Based on the results of these studies, DISERDIS is developed as a high fidelity prototype that addresses the requirements of the UCD phases, but is not yet a fully developed software system [20]. The system allows users to enter and view data of patients without a diagnosis trough a web application. The data includes demographic data such as diagnoses, symptoms, or information about the patient's family history. Additionally, the system allows the user to perform a patient similarity analysis by comparing data undiagnosed patients with previously diagnosed patients in different MIRACUM RDCs. The results of this similarity analysis are then visualized in different ways, such as scatterplots, patient-timeline views, or time-series charts. If one or more similar patients are identified, the user can search for experts on the particular disease using the “SE Atlas platform” (www.se-atlas.de).

As a supplement and continuation of our further work, DISERDIS should be evaluated to see to what extent it already meets user requirements [21]. Especially in CDSS, the evaluation of usability plays a decisive role. Usability refers to the quality of a system, which allows users to complete a task effectively, fast, and with high satisfaction [22]. While many studies propose methods to improve the performance and accuracy of diagnostic CDSS, to our knowledge, there is no study available that focuses on the design of a CDSS for RDs [17]. Moreover, we could not identify any study that assesses the usability and acceptance of a CDSS for RDs amongst users. Therefore, we performed an evaluation study of DISERDIS within the UCD, to discover improvable aspects of the system in order to increase user satisfaction. The objective of this study was to investigate whether the functionalities and information in DISERDIS were implemented in a user-friendly manner and to find out which functionalities of DISERDIS are perceived as particularly useful in order to derive recommendations for further optimization of the system.

## Methods

### Design

To investigate the usability of the software, a “Thinking-Aloud-Test” (TA-Test) was performed. This method was originally developed in the field of thinking psychology to record human cognitive processes. However, studies have shown its effectiveness in the evaluation of a software system [23–25]. “Thinking aloud” means that the users communicate their thoughts aloud while interacting with the software. During the interaction, the user indicates why they perform certain actions and what their goal is [26]. We have chosen this approach to get the opinions and feedback of all stakeholders in the MIRACUM consortium for our CDSS.

This study was conducted and performed according to the Consolidated Criteria for Reporting Qualitative Research (COREQ) [27, 28]*.* We considered 31 out of 32 items of COREQ in our study. A checklist is provided in Additional file: 1.

### Setting and sampling

In this study, we used a purposeful sampling, as in the previous UCD studies [18, 19]. Experts in the field of RDs, known by the authors, were invited for participation [29, 30]. According to Meuser and Nagel, an “expert” can be defined as a person with knowledge in a specific research area that is not accessible to everyone. Experts have experience and knowledge and can act upon their knowledge [30]. Experts were recruited from Rare Diseases Centres (RDCs) within the participating hospitals in the MIRACUM consortium. These centres are specialised facilities for patients without a diagnosis or RDs [31]. As in our former studies, we took the following characteristics of study participants into account: Participants are members of the MIRACUM consortium and work in a medical centre where specific RDs are diagnosed and treated (RDC). They have a completed medical degree and specialist training in human medicine. Considering these characteristics, eight potential study participants were identified, since eight of ten MIRACUM locations have established an RDC.

The participants were contacted by e-mail and asked to suggest a date and time. The invitation was sent in October, 2019. We distributed a study information letter to the study participants along with the email invitation.

### Data collection

#### Description of the test environment

The study was carried out under laboratory conditions, which means that DISERDIS was used in a test scenario and not in a real clinical scenario [32]. Ten fictitious patient cases were created together with an expert for RDs and used in the TA-Test and stored in the CDSS database [21, 33]. A description of the patient cases and functionality of the CDSS are shown in Additional files: 2 and 3.

#### Preparation and implementation of the study

An instruction sheet was created with three tasks that the participants were supposed to perform during the study (shown in Additional file: 4, translated from German to English) which also includes a patient case chosen out of the 10 factious patient cases. The study participants were asked to use the DISERDIS with a patient case of a 49 years old female patient, with different neurological symptoms and pneumological characteristics.

The instruction sheet was divided into two categories: (a) use of patient management and (b) use of the similarity analysis in the CDSS. The participants were ought to use the function of the similarity analysis of the CDSS, whereas the similarity analysis is calculated between the patient case, which is also shown on the sheet, and all other available cases in the database of the CDSS. Therefore, each study participant used the same patient case to have the same conditions for evaluation.

In addition to the instruction sheet, a moderation guide was prepared which served as an orientation for the test leader during the study containing questions to maintain the flow of a discussion with study participants [34].

Before the study, a pre-test was performed to evaluate the tasks, test instructions, and the time duration of the test session (43 min). After the pre-test, only minor changes to the instruction sheet and the course of the study were necessary.

The study was conducted by JS from November to December, 2019, whose research characteristics are the following: “gender: male”, “experience: 4 years research experience in medical informatics”, “degree: M.Sc. in Medical Informatics”, “occupation: research assistant and PhD student”.

The study was executed during the working hours of the study participants in their offices and the prototype of the CDSS was provided on a laptop. To avoid interruptions, no other persons were present during the study. After signing the consent forms, the instruction sheet was handed out to the participants and the test leader answered their questions. During the study, the on screen activities of the participants were recorded with the OBS-Studio software and an audio recording setting was recording their verbal statements. Once the TA-Test was completed, the audio and video recording were stopped and participants were provided with a questionnaire which is further described in the next section.

In summary, the TA-Tests lasted between 18 and 43 min, with an average duration of 30 min. The TA-Tests were conducted only once and were not repeated.

### Data analysis and processing

The analysis of the study was based on a qualitative content analysis according to Mayring [35] and a questionnaire. Qualitative content analysis is a structured, qualitative method for evaluating text-based data. The evaluation process is characterised by a rule-guided, fixed procedure [35]. In the following section, the qualitative content analysis approach and the used questionnaire are described in more detail.

#### Qualitative content analysis

A transcription protocol was created with Microsoft Word that includes the indication of the time in the video and audio recording. The statements of the study participants were transcribed into written form in the transcription protocol. The transcription was based on the transcription system of Kuckartz et al. [36]. Additionally, the user's interactions were described to trace which interactions the user performed in the software at any given time. A transcription protocol was created for each TA-Test.

**Fig. 1:**
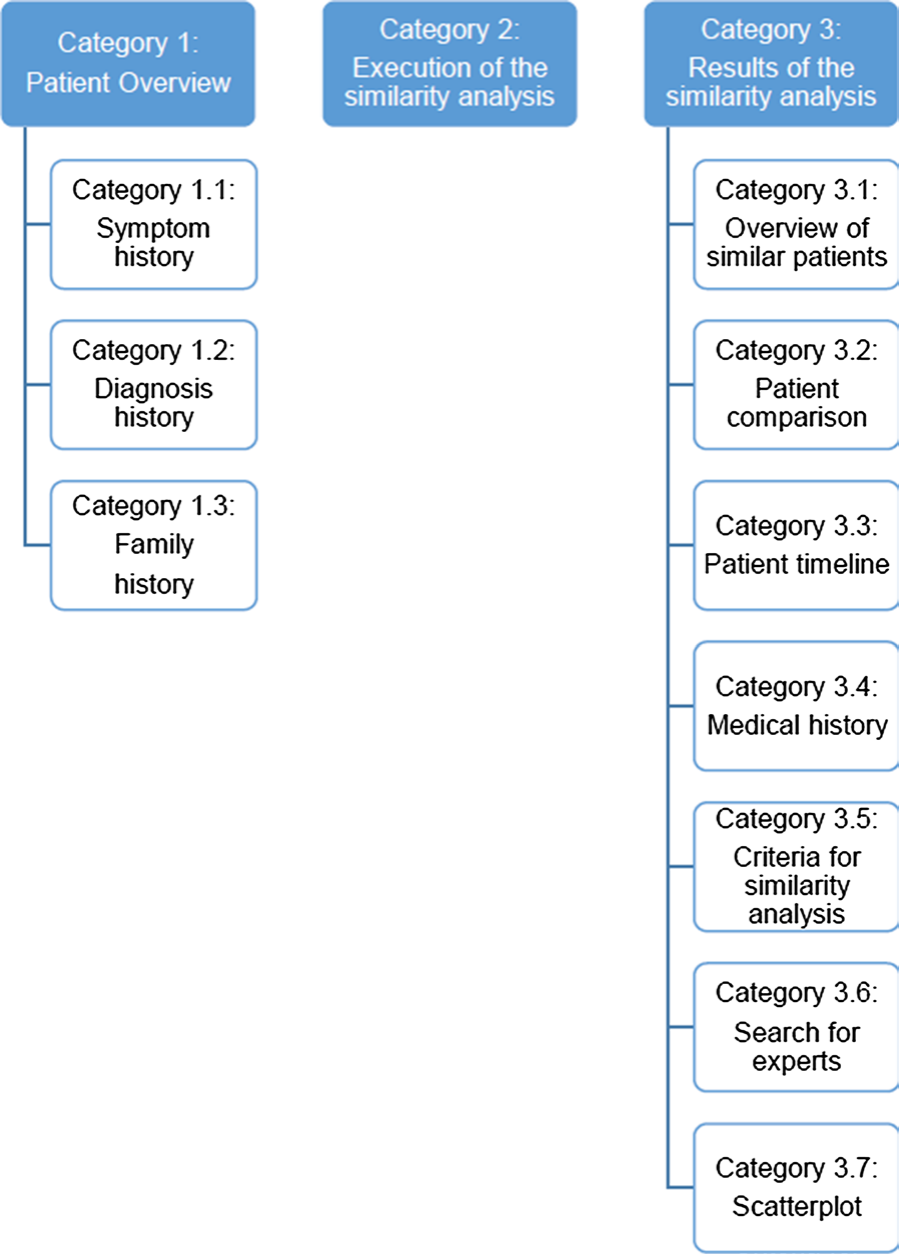
Final category system for content analysis

The transcripts were checked for validity and possible errors were corrected (e.g. missing words or sentences). The transcripts were returned to the study participants for validation, whereupon all participants confirmed the content of the transcripts. For data analysis of the transcripts, deductive categories were created to assign text passages from the transcripts to the categories [35]. Deductive categories are used to evaluate a qualitative content analysis and are defined before the study begins. They are divided into main-categories and sub-categories. The category system used in this study is based on the deductive categories, shown in Fig. 1. We defined the categories based on our research questions. To answer our research questions, all relevant software functions and user interfaces are available in the category system. For each category, the sub-categories “information”, "software functionality” and “usability” were also examined according to the research questions. These sub-categories are defined as follows:

- Information: The statements of the study participants refer to information presented in the CDSS. Information are presented after the use of a software function.
- Software functionality: The statements of the study participants refer to a software function of the CDSS.
- Usability: The statements of the study participants refer to the usability of the CDSS.

The transcribed material was proven in advance using the category system, to determine whether the categories can be applied to the data material. For this step, we used two (n = 2) transcription protocols, as it is recommended to use 10–50% of the transcribed material [37]. Afterwards, the category system was refined and two categories (1.3 and 3.7) were added to allow a more precise subdivision. After that, all transcripts were used and text passages were assigned to the categories. If a text passage could not be assigned to a category, all authors discussed and decided the assignment. Saturation of the study was reached when (1) all participants had successfully completed the study and (2) when the categories were adequately represented in the data after refinement of the category system [38].

While applying text passages to categories, we defined anchor examples and described when a text passage should be applied to a category. After the text passages were assigned to the categories, the respective statements were summarised per category. Finally, all results of the study were distributed to the study participants. All participants agreed to the results. In order to present the results in this paper, quotations were selected that represent the category at its best. The quotations (shown in Additional file: 5) were translated from German into English.

#### Questionnaire

For further analysis in the evaluation of the CDSS, we developed a questionnaire which consists of three parts. In part one, the System Usability Scale (SUS) was used, which is a standardised questionnaire with ten questions (items) to assess the usability of a software system [39]. Since the items are only available in English, they have been translated into German. In the second part of the questionnaire, further questions were created to evaluate the individual functionalities of the CDSS. These questions were answered using a 5-point Likert scale (from “1 = strongly disagree” to “5 = strongly agree”) [40]. In the third part of the questionnaire, participants were asked to provide some personal information [21]. The following information were collected: gender, age group, medical specialization, years of experience in the field of RDs and previous experience with CDSS.

For the data analysis of the SUS, we used the approach of Bangor et al. [39]. The result is a range from “0” to “100”, which isn’t supposed to be interpreted as a percentage and must be normalised. According to Bangor [41], normalisation can be achieved by using the range shown in Table 1. Furthermore, we calculated the mean and standard deviations for each item of the SUS across all participants.

**Table 1:** Usability ranges of the SUS according to Bangor [41]

SUS score (range)	Ranking
84.1–100.0	Best imaginable
80.8–84.0	Excellent
71.1–80.7	Good
51.7–71.0	OK
25.1–51.6	Poor
0.0–25.0	Worst imaginable

The answers to the questions in the second part of the questionnaire were assigned with numerical values from “1” to “5” according to the Likert scale, whereas mean and standard deviations were calculated for each question across all participants.

## Results

In this section, the results of the study are presented. First, we describe the characteristics of the study participants which is followed by the results of the qualitative content analysis and its categories. In the third part, the results of the questionnaire are described.

### Participants

All eight contacted experts responded positively to our invitation and participated in the study. We therefore achieved a participation rate of 100%. One study participant took part in the study, but due to time constraints, could not fill out the questionnaire, therefore we only have the data from seven participants. The characteristics of the study participants are shown in Table 2.

**Table 2:** Characteristics of study participants

Characteristics	Options	Participants (n = 7)
Gender	Female	4
	Male	3
Age group	> 59	1
	50–59	1
	40–49	2
	30–39	3
Medical specialization	Paediatric surgery	1
	Neurology	1
	Psychiatry and neurology	1
	Nephrology	1
	Internal medicine	1
	Neuropediatric	1
	Paediatric	1
Years of experience in the field of rare diseases	30	1
	24	1
	15	1
	4	2
	3	1
Prior experience with clinical decision support systems	Yes	4
	No	3

The participants were predominantly female (n = 4). The distribution of the age group was between 30 and 39 and older than 59 years. The study participants have different medical specialisations. It is noticeable that three study participants are working in paediatrics. The experience of the study participants in the field of RDs ranges from 3 to 30 years. In total, four of the study participants stated that they had already worked with any kind of CDSS.

### Main themes by deductive category

The results of the qualitative content analysis are presented in the following sections. Results are reported by deductive categories of the category system. We provide selected quotations for each statement (see Additional file: 5). The following exemplary quotes are abbreviated by “Q” and numbered in ascending order.

#### Patient overview (category 1)

##### Summary

For the Patient Overview (category 1), we discovered both positive and negative aspects. Participants stated that the patient information is displayed clearly and comprehensively. However, more detailed patient information can be helpful. Additionally, a calculation of the patient's age is also required.

##### Information

One study participant stated that the expected information about the patient was available in the patient’s overview (Q1). Two other study participants noted that more particular findings, such as doctor letters, laboratory or image findings are necessary to determine who diagnosed the RD (Q2-Q4).

In the diagnosis history and symptom history (category 1.1 and 1.2), the participants described the provided information as insufficient (Q5-Q8). One participant said: “*So, basically, it is not enough for me. But it depends on whether this is a first, what are the symptoms. Then of course it is sufficient. But in principle I would like to have details. In the case of dyspnoea in particular, this is high dyspnoea, dyspnoea caused by stress, and in case of fever, how high are the temperatures really, is this a chronic increased body temperature or fluctuating symptoms. The same applies to paraesthesia, i.e. which extremities are affected. Paraesthesia can have very different qualities. Depressiveness would suffice and fatigue, which are more descriptive. But this is not enough for me. But to have a first overview, then yes.”* (Q6).

Similarly, in the symptom and the diagnosis history section, the family history (category 1.3) was considered insufficient by most of the study participants and suggested a better illustration of family relationships (Q9). For example, one expert stated that the system lacks information on whether a family member had similar diagnoses or symptoms. A family tree could be a tool to illustrate family relationships and their illnesses (Q10-12). Another participant indicated that while consanguinity can be a marker for a genetic disorder, it is not relevant for all types of diseases (Q13). The participant stated: “*In that in the constellation as it exists right now, this is not my first question. Nevertheless, this is interesting, because whenever you have to make a diagnosis, it is not uninteresting to investigate consanguinity. Because paediatrics 80% are genetic. That is super interesting. But in the symptom complexes that were given here, that would not have been my first question.”* (Q13).

##### Usability

The participants agreed that the views of the patient overview, symptom history, diagnosis history and family history (category 1–1.3) are presented simply and clearly (Q14-17). One study participant explained: “*Otherwise, the mask is clearly arranged.*” (Q18).

##### Functionality

One of the participants stated that besides symptoms, the patient's age and the age at which the symptoms occur are important. The participant recommended calculating the age and the age at the onset of symptoms (Q19). One study participant suggested integration of a feedback function to enable the physician to mark the diagnoses as valid or invalid. This could e.g. indicate whether a diagnosis was made following medical guidelines (Q20).

#### Execution of the similarity analysis (category 2)

#### Summary

The results show that the views regarding the execution of the patient similarity analysis (category 2) should be improved. Furthermore, the computing time of the similarity analysis is described as too slow on the one hand and sufficient on the other hand.

##### Information

No data available for this category.

##### Usability

Before the similarity analysis, the user needs to select MIRACUM locations where data matching should take place. This icon is located in the lower section of the graphical design, which required quite some time for the participants to find (Q21-22). (Q21-22). Two study participants stated that the selection of all locations must be placed in the table headline (Q21-22).

##### Functionality

Two study participants made statements about the calculation time of the similarity analysis. One participant rated this process as too slow (Q23), while another participant stated: “*Well, that didn't take too long, 45 s. Maybe even 30.*” (Q24).

#### Results of the similarity analysis (category 3)

##### Summary

In performed similarity analysis (category 3), the date of birth was considered irrelevant. Additionally, information previewed in the medical history was rated as interesting and relevant. Regarding the usability, the overview of similar patients was rated positively. However, the search for an expert for RDs was perceived as non-intuitive. Moreover, the study participants criticised the lack of transparency of the similarity algorithm. It must be explained comprehensively, how the algorithm leads to the results.

##### Information

When presenting the overview of similar patients (category 3.1), the participants consider the date of birth irrelevant, but the age at the time of the diagnosis as highly relevant (Q25-26). The views of the patient timeline (category 3.3), medical history (category 3.4) and criteria for similarity analysis (category 3.5) also contain corresponding statements from participants (Q28-31). In addition, the patient comparison (category 3.2) should differentiate between the age of the patients and the age at the time of the diagnosis.

The information provided on the medical history (category 3.4) were rated as interesting and relevant (Q31-35). One study participant used an example to explain why the medical history is relevant for diagnosis: *“History is always very helpful. Especially in rare diseases. Because then only the history of the disease provides the information. If you think it's from my field, neurology, psychiatry, for example. With atypical Parkinson's syndromes. If you start with a cross-sectional approach, then you can hardly tell the difference at the bottom of the scale. But then the progression of certain symptoms, like the increase or decrease, especially the increase. This will basically give you the information about the diagnosis. As I mentioned earlier, this varies greatly from illness to illness.*” *(Q36).*

Apart from the advantages of such a view, one participant emphasised that the information in the medical history must be chosen in a way that it really helps to answer the question (Q37). One study participant stated that only those parameters should be displayed in the medical history that are specifically rare: “*I think something that is a common symptom of an extremely common disease, you don't need it here. Instead, these should be things that are specifically rare and therefore more specific.*” (Q38).

When searching for an expert for a diagnosis (category 3.6), two participants discussed which persons should be considered as an expert. They explained that an expert should have published at a high quality level (Q39-40).

##### Usability

The participants rated the usability of the overview of similar patients (category 3.1) positively (Q41-42). One participant stated: *“This is all very clear to me.”* (Q43). Additionally, the patient timeline (category 3.3) was also rated as useful (Q43-46). One participant suggested to include more than one patient in the patient timeline view (Q47).

One study participant stated that the comparison of two patients (category 3.2) is helpful (Q48). He proposes to place the demographic data at the beginning of the table (Q49).

Two participants could not find the function to set the criteria for the similarity analysis (category 3.5) (Q50-51). However, they noted that a regular use of the system may make it easier to use (Q52-53). Two study participants proposed to configure the criteria of the similarity analysis before the analysis is performed (Q54-55). One participant stated that the function to search for experts for a diagnosis (category 3.6) is not intuitive: “*No, not very intuitive. That what I did earlier. Oh, that was not related to the patient at all. Then it's perfectly fine. Then it is. You only have to know it once and then. Perfect. Okay.*” (Q56).

One participant had difficulties to find the scatterplot (category 3.7) (Q57). Another participant also suggested that more information should be displayed when you click on a point in the scatterplot (e.g. information on diagnosis) (Q57-58). One participant said that the benefits of the scatterplot occur when there are a large number of patients. However, he rated the tabular presentation as better, as it allows to view several pieces of information at a glance (Q59).

##### Functionality

The study participants criticised the lack of transparency of the similarity algorithm (category 3.2) (Q61-62). They explained that it must be comprehensible how the algorithm leads to the results (Q63-Q67). One participant stated: “*The displayed parameters. The anonymous patient was 63 years old when she was diagnosed. Similarity between the patients. Exactly here I miss again, which diagnosis have exactly matched?”* (Q67).

One participant rated the search for an expert in the CDSS as a good feature (category 3.6). However, he suggested that it should be possible to search for diagnoses that do not only refer to the confirmed diagnosis of a patient. The diagnoses of the diagnosis history should also be included in the search (Q68). One participant noted that it would be helpful to include differential diagnoses in the system and to include them in the search (Q69).

Regarding the scatterplot (category 3.7), one participant suggested to use a spider chart to plot the individual components, such as symptoms, diagnoses and family history (Q70).

### Results of the questionnaire

The first part of the questionnaire (questions 1–10), which is related to the SUS, resulted in a score of “73.21”. Thus, the CDSS achieved the usability rating “good”, according to Bangor et al. [39]. More details are shown in Table 3.

**Table 3:** Results of the system usability scale (SUS)

SUS-item	Question	N (valid)	Mean	SD
1	I think that I would like to use this system frequently	7	3.85	0.9897
2	I find the system unnecessarily complex	7	1.71	0.4518
3	I thought the system was easy to use	7	3.71	0.4518
4	I think that I need the support of a technical person to be able to use this system	7	2.57	0.4949
5	I found the various functions in this system were well integrated	7	3.85	0.8330
6	I thought there was too much inconsistency in this system	7	2	0.5345
7	I would imagine that most people would learn to use this system very quickly	7	4.57	0.4949
8	I found the system very cumbersome to use	7	2	0
9	I felt very confident using the system	7	3.71	1.1606
10	I needed to learn a lot of things before I could get going with this system	7	2.14	0.8330
Overall SUS score	73.21			

n = 7 participants, mean rating (5-point scale strongly disagree = 1; disagree = 2; neutral = 3; agree = 4; strongly agree = 5), standard deviations (SD) and overall SUS score

The second part of the questionnaire (questions 11–21) with specific items to individual functionalities resulted in a rating between 3.42 and 4.28. Thus this correspond to the characteristic value from “neutral” to “agree”, according to the 5-point Likert scale. More detailed results of the questionnaire are shown Additional file: 6.

## Discussion

This qualitative study investigated the usability of a CDSS for RDs. In concrete terms the study investigated whether the functionalities and information in the CDSS were implemented in a user-friendly manner and which changes are necessary to improve the CDSS.

### Discussion of results

Developing a CDSS specifically for the application area of RDs involves specific challenges: Physicians have a special need for information, the decision proposals must be communicated to the physician in a transparent and comprehensible manner, the system must be intuitively usable, and only a few experts in this field are available for user-centred development and evaluation. For this reason, the evaluation was conducted via expert interviews with a focus on the aspects of information adequacy, usability, and functionality of the CDSS. In the following sections, these three aspects are discussed in more detail.

### Information

With regard to the information shown in the patient cases in the CDSS, the study participants stated that relevant clinical information and findings are missing in order to evaluate a patient case more accurately. For example, more detailed descriptive information for symptoms, diagnoses and family histories are required. Participants need more information about the findings related to a diagnosis and about the corresponding physician who has diagnosed the patient. In terms of the family history, relationships and information about family members of the patient should be provided. Hence, there needs to be a revision about what kind of information of patient cases are presented. This finding is similar to the results of other studies which indicate, that the success of CDSS is independent from the consistency of the knowledge base, data quality and content [12, 42].

However, since RDs are very heterogeneous and a general data set that describes all diseases cannot be defined, it might be possible to display parameters by disease groups [19]. However, the development of a data set by disease groups depends on what patient data is available at the MIRACUM locations. This should be investigated in further studies before the CDSS is used in clinical practice.

Another aspect mentioned in the context of the information displayed in the CDSS is that the date of birth of a patient is not relevant and the age, e.g. when a symptom occurred, should be calculated.

Despite these limitations of the information presented in the CDSS, the study participants rated some views, such as medical history, as helpful and relevant. This is consistent with the results of the questionnaire. However, the clinical parameters presented there have to be selected in such a way that they really help to answer the question for the patient.

### Usability

The results concerning usability show that there is an acceptance of the clinical users towards the CDSS. This is confirmed by the results of the SUS, what indicates that the usability of the CDSS was rated as good. However, the results may have been influenced by the fact that the information on the patient cases were insufficient for the study participants. It is possible that acceptance to the system will increase, if this aspect is addressed in further developments. However, most of the study participants stated that they could imagine to use the system in the future. Furthermore, the views for the symptoms, diagnoses, family history and the overview of similar patients were rated as structured and clear. In addition, the patient timeline and the comparison of two patients were rated as useful.

The study also identified usability problems. For instance, one problem refers to the software function to execute the similarity analysis. The users were not able to select all MIRACUM locations as stated in the instruction sheet. However, the study participants indicate that once you have found the function, you know how to use it. Another usability problem was identified in the function of the set criteria for the similarity analysis, since two study participants were not able to find the function in the system.

### Functionality

Regarding the software functionality of the CDSS, participants stated that they could easily determine which patients are the most similar and could quickly view demographic information. These results were also available in the questionnaire. In the overview of similar patients, the participants stated in the TA-Test as well as in the questionnaire, that the tabular presentation is favoured over the scatterplot. Furthermore, the participants suggested some improvements for the CDSS. For example, it should be possible to display several patients in the patient history. Additionally, the participants indicated that a sufficient transparency in the results of the similarity analysis must be available. This is necessary in order to determine which symptoms or diagnoses match exactly. Other studies also describe the transparency of a CDSS as an essential success factor for acceptance [43–45].

The search for an expert for RDs was considered as an important feature. However, we could not identify any other CDSS study that evaluated a similar function. In the future, the search should make it possible to search not only for the established diagnosis of the similar patient, but also for differential diagnoses.

### Summary

As the study has revealed some advantages and disadvantages, the optimization of the CDSS should include the following aspects:

- The CDSS should include more detailed information about patients.
- The CDSS should calculate the age of the patient where it is necessary.
- The view of the patient history should include several patients.
- A higher transparency in the results of the similarity analysis is needed (e.g. which symptoms were similar).
- The search for an expert for RDs should allow to search for any diagnosis, not only for the established diagnosis of the similar patient.
- Usability issues should be solved, like the selection of all MIRACUM locations or to find the function of set criteria for similarity analysis.

### Discussion of methods

In this study, we have chosen a TA-Test in combination with the SUS, which is a common approach to evaluate the usability of a CDSS [24–26, 46]. Furthermore, the TA-Test is easy to implement and allows us to primarily assess usability weaknesses as well as the functionality and information of the CDSS in an early stage of the development [25]. However, acceptance models like the Technology Acceptance Model (TAM) could be used in the future, which focus more on implementation and the intention to use and less specifically on positive and negative design aspects of a system. They are therefore less suitable for deriving concrete re-design suggestions and more for identifying implementation barriers. Therefore, it could be of interest in a later version of our system [47].

As an alternative methodology to the TA-Test, so-called “Near Live Clinical Simulations” (NLCS) could be used [48]. For example, Li et al. used NLCS to evaluate their CDSS, in which the participants are in a prepared treatment room similar to in clinical routine. The study participants are confronted with different case scenarios, at which the patient cases are simulated by actors and they are recorded on video tape. The authors concluded that the use of NLCS provides results on how the clinical workflow affects the use of the CDSS [25]. As our CDSS was still in an early phase of development, we did not opt this method due to the high effort required for evaluation. However, this method could be an opportunity to investigate the CDSS in a more realistic clinical scenario after the refinement of the system. To reduce the high effort in preparation time and the evaluation in NLCS, the “Instant Data Analysis” (IDA) can be used [46]. The IDA is supposed to reduce the amount of work and time needed for the execution and analysis of a usability test. In IDA, several individual sessions are conducted on one day. At the end of the sessions, the study participants meet to discuss the identified usability problems [46]. Both IDA and NLCS could be ways to further evaluate and refine the CDSS within the UCD.

### Limitations

The study has several limitations. The results of the study may have been affected by the presence of a test leader throughout the test scenario. This could have an impact on the natural behaviour of the participants, as they know they are under observation (Hawthorne effect) [24]. Due to the fact that the experts were known to the author, it cannot be excluded that there is a selection bias or that answering behaviour in the sense of "(social) desirability" occurred.

Another restriction is that the results are limited to the RDCs in the MIRACUM consortium. More RDCs could be included in a further study. Currently 33 RDCs are available in Germany [49]. Like in our expert’s interview study and any other qualitative study, qualitative research is not intended to produce representative and generalizable results. Nevertheless, using a purposeful sampling is common in qualitative research and, according to the literature, 8 participants are sufficient since 8–10 participants can identify 80% of the usability problems of a software [33, 40, 41].

Another limitation is that the system was only evaluated under laboratory conditions. No real patient data was used and the system was not provided on the participant's own computer. Using only one patient case, which was created by an expert for RDs, could have an impact on the results. It could be possible that the experts give different opinions when using different patient cases. Therefore, as mentioned, follow up studies are necessary.

Nevertheless, a lab test offers advantages, e.g. the conditions are controllable and the results are reproducible. A further limitation was that no data are available for the subcategory "Information" in category 2 "Execution of the similarity analysis". Moreover, one participant did not complete the questionnaire of the study. Nevertheless, using a high methodological standard with COREQ could minimize possible bias across the study.

## Conclusion

This qualitative study involved experts for RDs to assess whether the functionalities and information in the CDSS were implemented in a user-friendly manner and which changes should be derived.

The results indicate that more details regarding information of patients are needed. Furthermore, most of the software functionalities were rated positively, whereas the study participants suggested some improvements for the functions. For instance, the transparency of the results provided by the CDSS for decision support are insufficient and should be refined.

Overall, the CDSS achieved a good usability score and most participants could imagine using the system in the future. Therefore, the developed prototype has potential to be used in the clinical practice. However, further work and studies are necessary to refine the CDSS and address the results and suggestions of this study. For the future, it remains interesting to evaluate the system again when it is used in clinical practice with real patient cases.

## Supplementary Information

**Additional file 1.** COREQ Checklist.

**Additional file 2.** Description of patient cases.

**Additional file 3.** Functionality of the CDSS.

**Additional file 4.** Instruction sheet.

**Additional file 5.** Example quotations of study participants.

**Additional file 6.** Results of the questionnaire.

## Data Availability

The datasets used and/or analyzed during the current study are available from the corresponding author on reasonable request.

## Abbreviations

BMBF: German Ministry of Education and Research
CDSS: Clinical Decision Support System
COREQ: Consolidated Criteria for Reporting Qualitative Research
DISERDIS: Diagnosis Support in Rare Diseases
IDA: Instant Data Analysis
MIRACUM: Medical Informatics in Research and Medicine
NLCS: Near Live Clinical Simulations
RD: Rare Diseases
RDC: Rare Diseases Centre
SUS: System Usability Scale
TAM: Technology Acceptance Model (TAM)
TA-Test: Thinking Aloud Test
UCD: User-Centred Design Process
WHO: World Health Organization
