# Supplementary material for: Evaluation of a clinical decision support system for rare diseases: a qualitative study
Source: BMC Med Inform Decis Mak. 2021 Feb 18;21:65. doi: 10.1186/s12911-021-01435-8 (PMC7890997; doi:10.1186/s12911-021-01435-8)
Supplement: Supplementary file 2 — Additional file 2. Description of patient cases. [file 12911_2021_1435_MOESM2_ESM.pdf]

Description of patient cases

Patient 1:

First name: Annette

Name: Baumgartner

Birth date: 23.04.1997

Gender: Female

Symptom history

Symptom	Symptom-Code (HPO)	Month of first occurrence	Year of first occurrence
Visual loss	HP:0000572	January	2017
Fatigue	HP:0012378	October	2016
Paraesthesia	HP:0003401	January	2019
Foot dorsiflexor weakness	HP:0009027	March	2019

Diagnosis history

Diagnosis	Diagnosis-Code (ICD-10)	Date of diagnosis	Diagnostic confidence	Time of occurrence
Optic neuritis	H46	20.02.2017	Confirmed	Not clear
Lesion of lateral popliteal nerve	G57.3	14.01.2019	Confirmed	Not clear
Lumbar and other intervertebral disc disorders with myelopathy	M51.0 +	14.01.2019	Suspected	Not clear
Acute maxillary sinusitis	J01.0	03.08.2012	Confirmed	Not clear
Headache	R51	14.09.2013	Confirmed	Not clear
Acute upper respiratory infection, unspecified	J06.9	03.12.2013	Confirmed	Not clear

Family history

Are the patient's parents blood relatives? No confirmed

Final Diagnosis

Diagnosis: Multiple Sclerosis

Diagnosis-Code: G35

Date of diagnosis: 15.03.2019

Diagnostic confidence: Confirmed

Rare disease: No

Patient 2:

First name: Fiona

Name: Bergmann

Birth date: 11.02.1970

Gender: Female

Symptom history

Symptom	Symptom-Code (HPO)	Month of first occurrence	Year of first occurrence
Dyspnoea	HP:0002094	January	2019
Fatigue	HP:0012378	December	2018
Fever	HP:0032324	December	2018
Depressivity	HP:0000716	January	2019
Paraesthesia	HP:0032120	March	2018

Diagnosis history

Diagnosis	Diagnosis-Code (ICD-10)	Date of diagnosis	Diagnostic confidence	Time of occurence
Acute upper respiratory infection, unspecified	J06.9	03.11.2016	Confirmed	Not clear
Predominantly allergic asthma	J45.0	15.12.2016	Confirmed	Not clear
Acute bronchiolitis, unspecified	J20.9	10.12.2016	Confirmed	Not clear

Family history

Are the patient's parents blood relatives? No confirmed

Final Diagnosis

Diagnosis: Antisynthetase syndrome

Diagnosis-Code: Orpha:81

Date of diagnosis: 09.09.2019

Diagnostic confidence: Suspected

Rare disease: Yes

Patient 3:

First name: David

Name: Schwarz

Birth date: 14.02.1988

Gender: Male

Symptom history

Symptom	Symptom-Code (HPO)	Month of first occurrence	Year of first occurrence
Night sweats	HP:0030166	August	2019
Weight loss	HP:0001824	July	2019
Fever	HP:0001945	August	2019
Lymphadenopathy	HP:0002716	July	2019

Diagnosis history

Diagnosis	Diagnosis-Code (ICD-10)	Date of diagnosis	Diagnostic confidence	Time of occurrence
Acute bronchitis due to Mycoplasma pneumonia	J20.0	09.09.1998	Confirmed	Not clear
cute upper respiratory infection, unspecified	J06.9	08.11.2000	Confirmed	Not clear
Low back pain	M54.5	09.07.2014	Confirmed	Not clear
Other specified intervertebral disc displacement	M51.2	18.07.2014	Confirmed	Not clear

Family history

Are the patient's parents blood relatives? No confirmed

Final Diagnosis

Diagnosis: Hodgkin lymphoma, unspecified

Diagnosis-Code: C81.9

Date of diagnosis: 10.09.2019

Diagnostic confidence: Confirmed

Rare disease: No

Patient 4:

First name: Phillip

Name: Neumann

Birth date: 23.08.1952

Gender: Male

Symptom history

Symptom	Symptom-Code (HPO)	Month of first occurrence	Year of first occurrence
Dyspnea	HP:0002094	January	2019
Chest pain	HP:0100749	October	2018
Palpitations	HP:0001962	February	2019
Vertigo	HP:0002321	January	2019

Diagnosis history

Diagnosis	Diagnosis-Code (ICD-10)	Date of diagnosis	Diagnostic confidence	Time of occurrence
Essential (primary) hypertension	I10.0	09.03.2009	Confirmed	Not clear
Mixed hyperlipidaemia	E78.2	04.03.2012	Confirmed	Not clear
Primary arthrosis of first carpometacarpal joints, bilateral	M18.0	18.04.2017	Confirmed	Not clear
Hyperuricaemia without signs of inflammatory arthritis and tophaceous disease	E79.0	05.06.2016	Confirmed	Not clear
Spondylosis, unspecified	M47.9	02.08.2000	Confirmed	Not clear
Fatty (change of) liver, not elsewhere classified	K76.0	10.03.2012	Confirmed	Not clear

Diagnosis	Diagnosis-Code (ICD-10)	Date of diagnosis	Diagnostic confidence	Time of occurrence
Coxarthrosis	M16.1	02.09.2018	Confirmed	Not clear

Family history

Are the patient's parents blood relatives? No, confirmed

Final Diagnosis

Diagnosis: Atherosclerotic heart disease

Diagnosis-Code: I25.1

Date of diagnosis: 06.06.2019

Diagnostic confidence: Confirmed

Rare disease: No

Patient 5:

First name: Leon

Name: Neustadt

Birth date: 23.08.1952

Gender: Male

Symptom history

Symptom	Symptom-Code (HPO)	Month of first occurrence	Year of first occurrence
Dyspnoea	HP:0002094	January	2019
Chest pain	HP:0100749	October	2018
Palpitations	HP:0001962	February	2019
Vertigo	HP:0002321	January	2019

Diagnosis history

Diagnosis	Diagnosis-Code (ICD-10)	Date of diagnosis	Diagnostic confidence	Time of occurrence
Essential (primary) hypertension	I10	09.03.2016	Confirmed	Not clear
Obesity, unspecified	E66.9	12.04.2000	Confirmed	Not clear

Diagnosis	Diagnosis-Code (ICD-10)	Date of diagnosis	Diagnostic confidence	Time of occurrence
Hyperuricaemia without signs of inflammatory arthritis and tophaceous disease	E79.0	05.06.2016	Confirmed	Not clear
Radiculopathy	M54.5	04.06.2014	Confirmed	Not clear
Other primary gonarthrosis	M17.1	15.04.2015	Confirmed	Not clear
Acute upper respiratory infection, unspecified	J06.9	03.12.2013	Confirmed	Not clear

Family history

Are the patient's parents blood relatives? No, confirmed

Final Diagnosis

Diagnosis: Type 2 diabetes

Diagnosis-Code: E11

Date of diagnosis: 15.07.2019

Diagnostic confidence: Confirmed

Rare disease: No

Patient 6:

First name: Marko

Name: Lehrer

Birth date: 04.04.1989

Gender: Männlich

Symptom history

Symptom	Symptom-Code (HPO)	Month of first occurrence	Year of first occurrence
Myalgia	HP:0003326	September	2015
Paraesthesia	HP:0032120	September	2015
Chronic Fatigue	HP:0012432	September	2015
Memory Impairment	HP:0002354	September	2015

Symptom	Symptom-Code (HPO)	Month of first occurrence	Year of first occurrence
Decreased female libido	HP:0030018	September	2015

Diagnosis history

Diagnosis	Diagnosis-Code (ICD-10)	Date of diagnosis	Diagnostic confidence	Time of occurrence
Sarcoidosis of lung with sarcoidosis of lymph nodes	D86.2	04.09.2014	Confirmed	Not clear
Carpal tunnel syndrome	G56.0	06.06.2015	Confirmed	Not clear
Primary biliary cirrhosis	K74.3	02.02.2016	Confirmed	Not clear
Other specified inflammatory liver diseases	K75.8	06.06.2015	Confirmed	Not clear
Depression and anxiety	F41.2	05.07.2019	Confirmed	Not clear

Family history

Are the patient's parents blood relatives? No, confirmed

Final Diagnosis

Diagnosis: Primary biliary cirrhosis

Diagnosis-Code: K74.3

Date of diagnosis: 05.07.2019

Diagnostic confidence: Confirmed

Rare disease: Yes

Patient 7:

First name: Swen

Name: Friedmann

Birth date: 13.11.2013

Gender: Male

Symptom history

Symptom	Symptom-Code (HPO)	Month of first occurrence	Year of first occurrence
Delayed speach and language development	HP:0000750	November	2015
Developmental regression	HP:0002376	May	2016
Global development delay	HP:0001263	March	2017

Diagnosis history

Diagnosis	Diagnosis-Code (ICD-10)	Date of diagnosis	Diagnostic confidence	Time of occurrence
Hypermetropia	H52.0	02.02.2017	Confirmed	Not clear
Astigmatism	H52.2	02.02.2017	Confirmed	Not clear

Family history

Are the patient's parents blood relatives? No, suspected

Final Diagnosis

Diagnosis: Monosomy 22q13.3

Diagnosis-Code: Orpha:48652

Date of diagnosis: 01.07.2019

Diagnostic confidence: Confirmed

Rare disease: Yes

Patient 8:

First name: Kathrin

Name: Berg

Birth date: 24.01.1955

Gender: Female

Symptom history

Symptom	Symptom-Code (HPO)	Month of first occurrence	Year of first occurrence
Nonproductive cough	HP:0031246	August	2017
Eczema	HP:0000964	August	2017
Tachypnea	HP:0002789	September	2017
Fever	HP:0001945	March	2018
Myalgia	HP:0003326	March	2018
Paraesthesia	HP:0032120	March	2018

Diagnosis history

Diagnosis	Diagnosis-Code (ICD-10)	Date of diagnosis	Diagnostic confidence	Time of occurrence
Allergic rhinitis due to pollen	J30.1	09.10.1980	Confirmed	Not clear
Predominantly allergic asthma	J45.0	10.08.2016	Confirmed	Not clear
Carpal tunnel syndrome	G56.0	01.05.2013	Confirmed	Not clear
Struma nodosa	E01.1	09.10.1985	Confirmed	Not clear

Family history

Are the patient's parents blood relatives? No, confirmed.

Final Diagnosis

Diagnosis: Antisynthetase syndrome

Diagnosis-Code: Orpha:81

Date of diagnosis: 01.05.2018

Diagnostic confidence: Confirmed

Rare disease: Yes

Patient 9:

First name: Sven

Name: Traugott

Birth date: 25.12.1984

Gender: Male

Symptom history

Symptom	Symptom-Code (HPO)	Month of first occurrence	Year of first occurrence
Weight Loss	HP:0001824	September	2016
Fatigue	HP:0012378	September	2016
Fever	HP:0001945	January	2017

Diagnosis history

Diagnosis	Diagnosis-Code (ICD-10)	Date of diagnosis	Diagnostic confidence	Time of occurrence
Acute hepatitis B without delta-agent and without hepatic coma	B16.9	06.06.2018	Confirmed	Not clear

Family history

Are the patient's parents blood relatives? No, confirmed.

Final Diagnosis

Diagnosis: Acute hepatitis B without delta-agent and without hepatic coma

Diagnosis-Code: B16.9

Date of diagnosis: 06.06.2008

Diagnostic confidence: Confirmed

Rare disease: Not clear

Patient 10:

First name: Anton

Name: Fuchs

Birth date: 08.05.1941

Gender: Male

Symptom history

Symptom	Symptom-Code (HPO)	Month of first occurrence	Year of first occurrence
Hypertonia	HP:0001276	January	2018
Memory impairment	HP:0002354	August	2018
Visual impairment	HP:0000505	June	2018

Diagnosis history

Diagnosis	Diagnosis-Code (ICD-10)	Date of diagnosis	Diagnostic confidence	Time of occurrence
Hyperplasia of prostate	N40	03.07.2011	Confirmed	Not clear
Complicated migraine	G43.3	09.03.1988	Confirmed	Not clear

Family history

Are the patient's parents blood relatives? No, confirmed.

Final Diagnosis

Diagnosis: Manganese poisoning

Diagnosis-Code: ORPHA:306682

Date of diagnosis: 01.12.2018

Diagnostic confidence: Suspected

Rare disease: Yes
