# Supplementary material for: Evaluation of a clinical decision support system for rare diseases: a qualitative study
Source: BMC Med Inform Decis Mak. 2021 Feb 18;21:65. doi: 10.1186/s12911-021-01435-8 (PMC7890997; doi:10.1186/s12911-021-01435-8)

Functionality of the CDSS

The following document describes the software functions of the CDSS. As the system is only available in German language, the individual views are described in detail and the translations of the respective information are given in brackets.

Overview and entry of patient data

Form fields for the entry of patient's data are provided. This includes entering the patient's master data, symptom history, diagnosis history, family history and final diagnosis. The following figure shows the form for entering the patient's master data. The patient's first name (Vorname) and last name (Nachname), date of birth (Geburtsdatum) and a unique identifier (Internes Kennzeichen) must be entered. This can be a unique number from a source system (e.g. a patient number from electronic health records). In addition, the patient can be prioritised (Priorität) between “high”, “medium” and “low”.

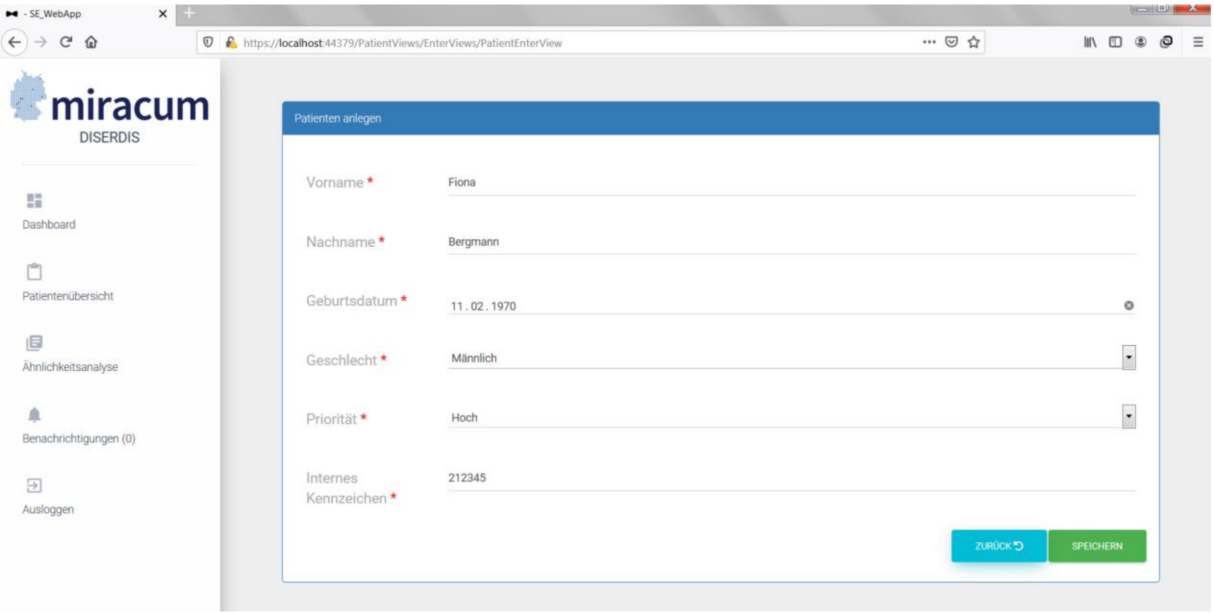

The following figure shows the overview of all patients present in the system at one MIRACUM location. For each patient, the name (Nachname, Vorname), date of birth (Geburtsdatum), diagnosis status (Diagnosestatus) and priority (Priorität) are shown. Furthermore, buttons are integrated to “perform a similarity analysis (Ähnlichkeitsanalyse durchführen)”, “view patient details (Patienten Details)” or “delete patient data (Patient löschen)”.

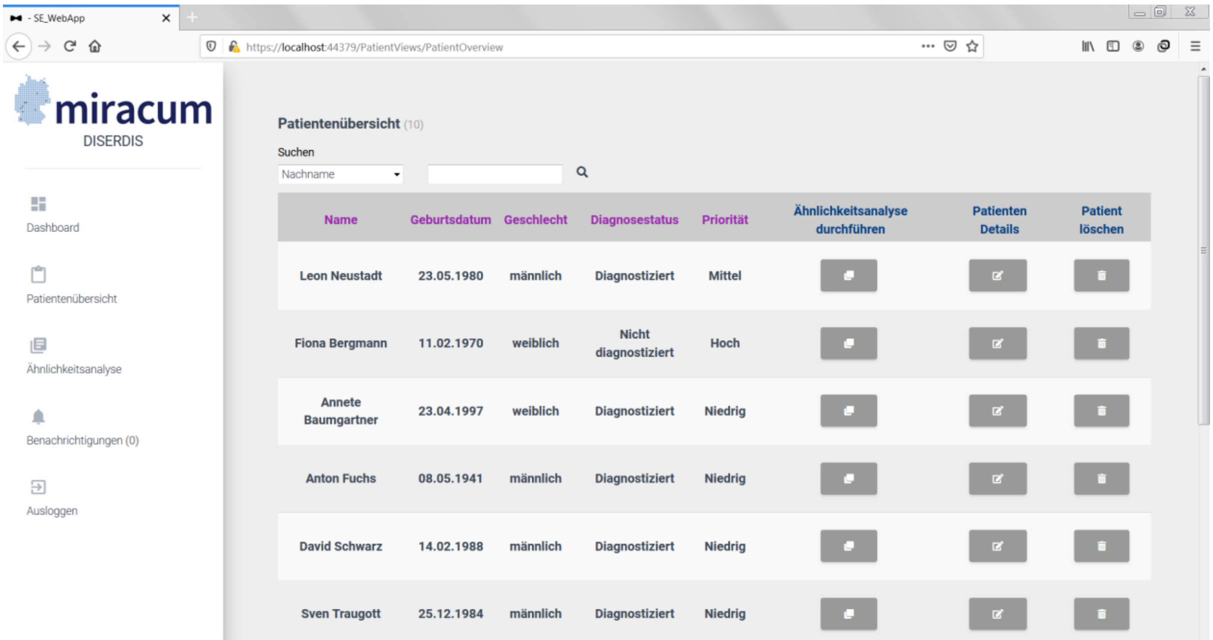

With a click on the “Patient details (Patienten Details)” button, the following view in the next figure is shown. This individual view of a patient allows further navigation to the patient's symptom history (Symptomhistorie), diagnosis history (Diagnosenhistorie), family history (Familienhistorie) or patient master data (Stammdaten). It is also possible to be forwarded to the “Final diagnosis (Finale Diagnose)” view. In addition, the results of the similarity analysis of the patient can be viewed by clicking on "Results of the similarity analysis (Ergebnisse der Ähnlichkeitsanalyse)”.

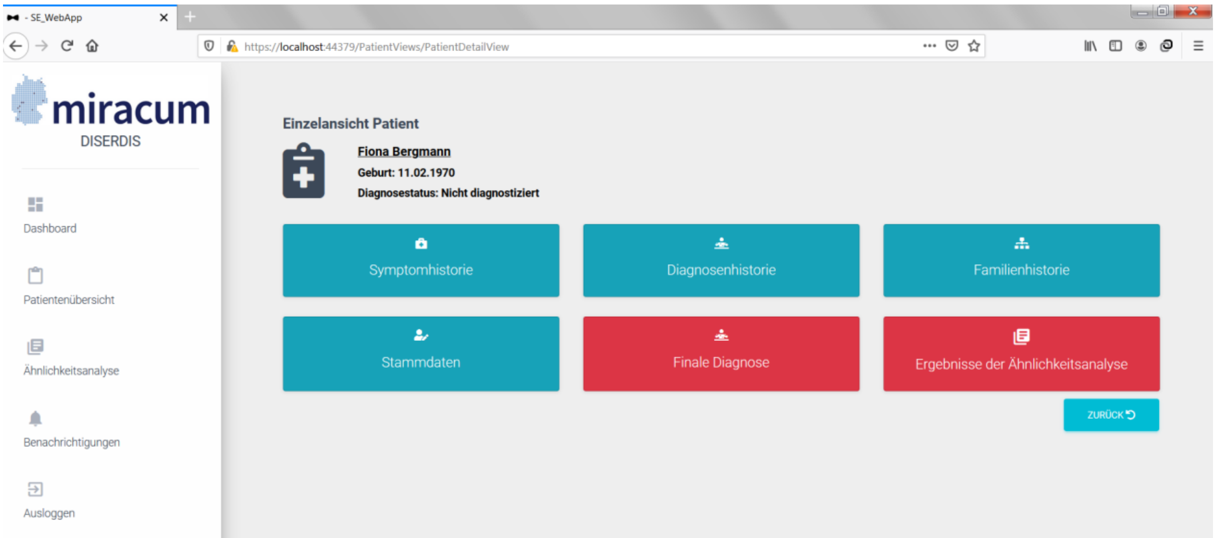

Selecting the patient's “symptom history” brings up the view shown in the following figure. In this, all existing symptoms of the patient, which are coded with the Human Phenotype Ontology (HPO), are indicated. The month and year, in which the symptom appears (Monat des ersten Auftretens, Jahr des ersten Auftretens), are also available.

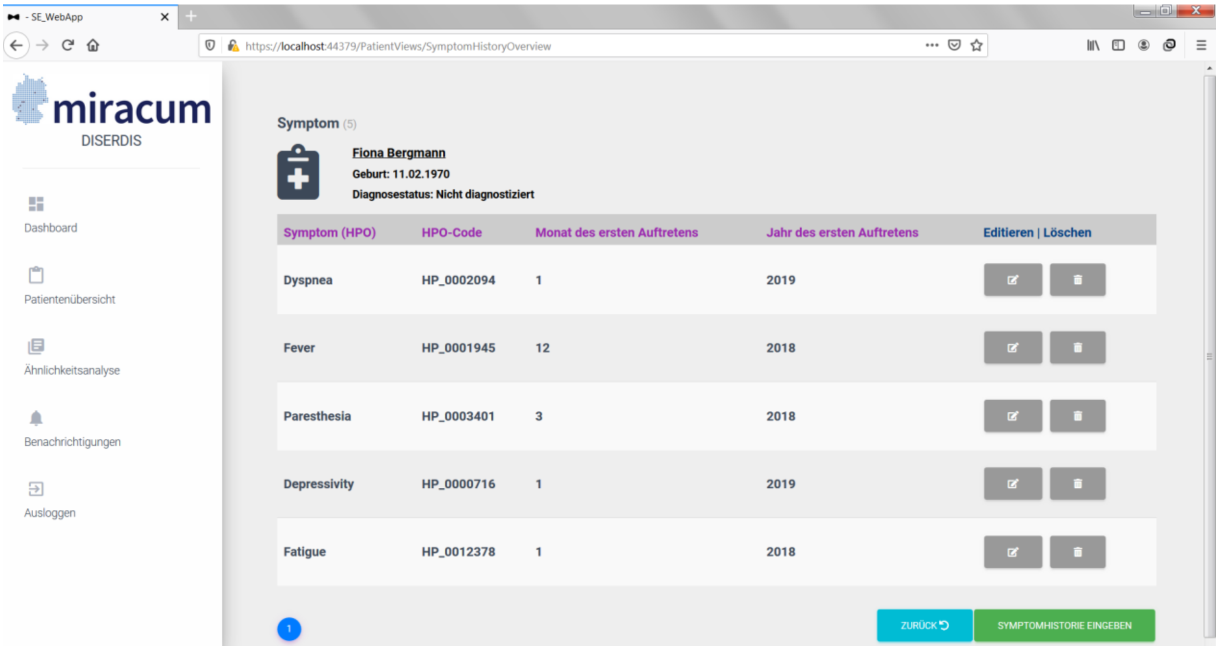

To enter a symptom for a patient, the button "Enter symptom history (Symptomhistorie eingeben)” can be clicked. With a click on "Edit (Editieren)” already entered data can be edited. The "Delete (Löschen)” button allows to delete a symptom. Selecting "Enter symptom history" displays the form shown in the figure below.

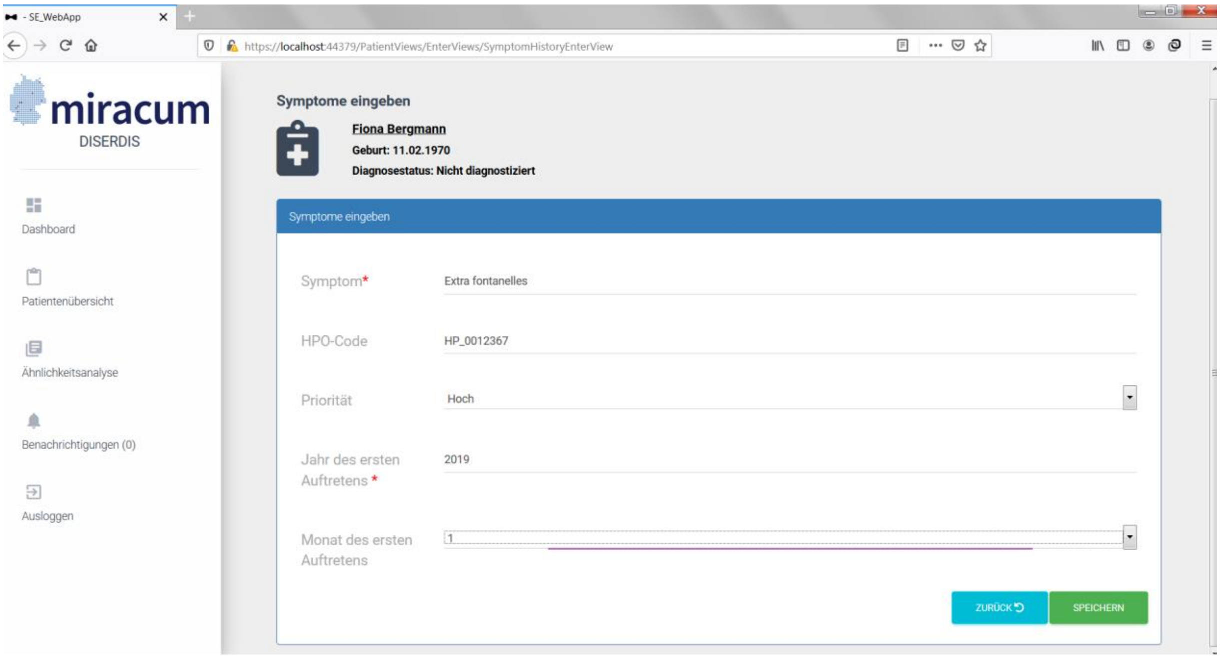

Several fields are available in the input form of the symptom history. Some of them are mandatory fields, which are marked with a red star. There are also optional fields that do not have to be filled. The scheme of mandatory and optional fields are present in all input forms in the CDSS.

The user has to enter the text of the symptom in a text-field and the corresponding "HPO code" is automatically selected by this system. The user is assisted by an automatic completion function. When the user types the beginning of a term, all terms that match the entry are

displayed. After the symptom is coded, it is possible to prioritize (Priorität) the symptom by specifying "High", "Medium" or "Low", thus weighting the symptoms. Furthermore, the indication in which year the symptom occured (Jahr des ersten Auftretens) is mandatory. The month of occurrence (Monat des ersten Auftretens) in the year is optional.

The following figure shows an overview of the diagnosis history. It shows the ICD-10 code, the diagnosis description (Diagnosebeschreibung), diagnosis date (Diagnosedatum) and the "Edit (Editieren)" and "Delete (Löschen)" buttons.

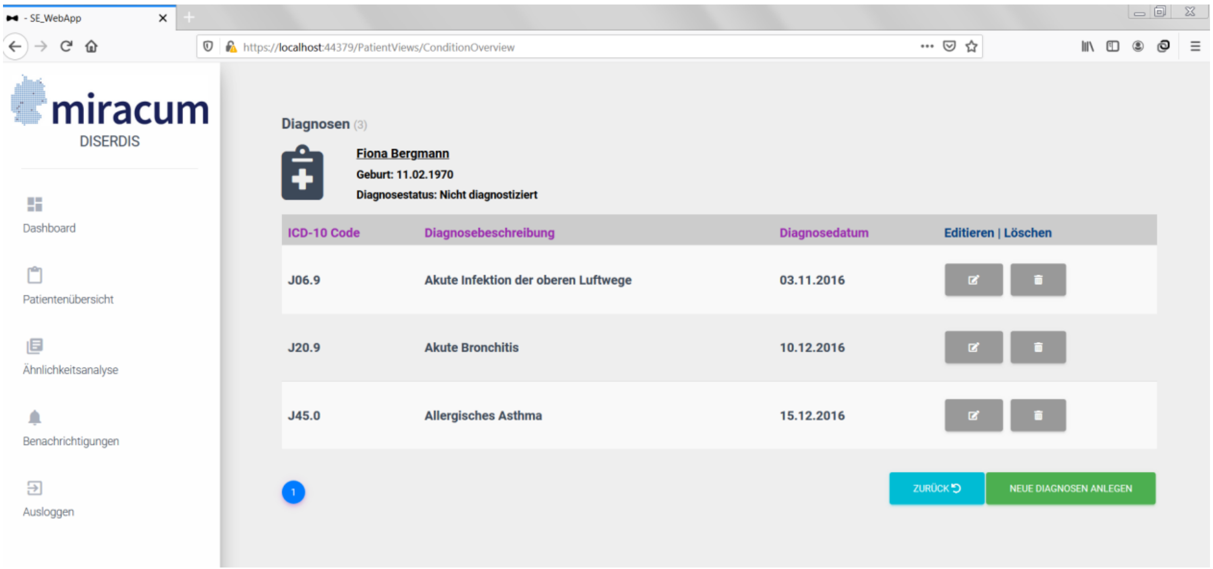

Similar to the symptom history view, the diagnosis can be entered via text, as shown in the following figure. The user is supported by an automatic completion function. The diagnosis (Diagnose) and the diagnosis date (Diagnosedatum) are mandatory fields. The diagnosis reliability (Diagnosesicherheit) as well as the time of occurrence of the diagnosis (Zeitpunkt des ersten Auftreten) are optional.

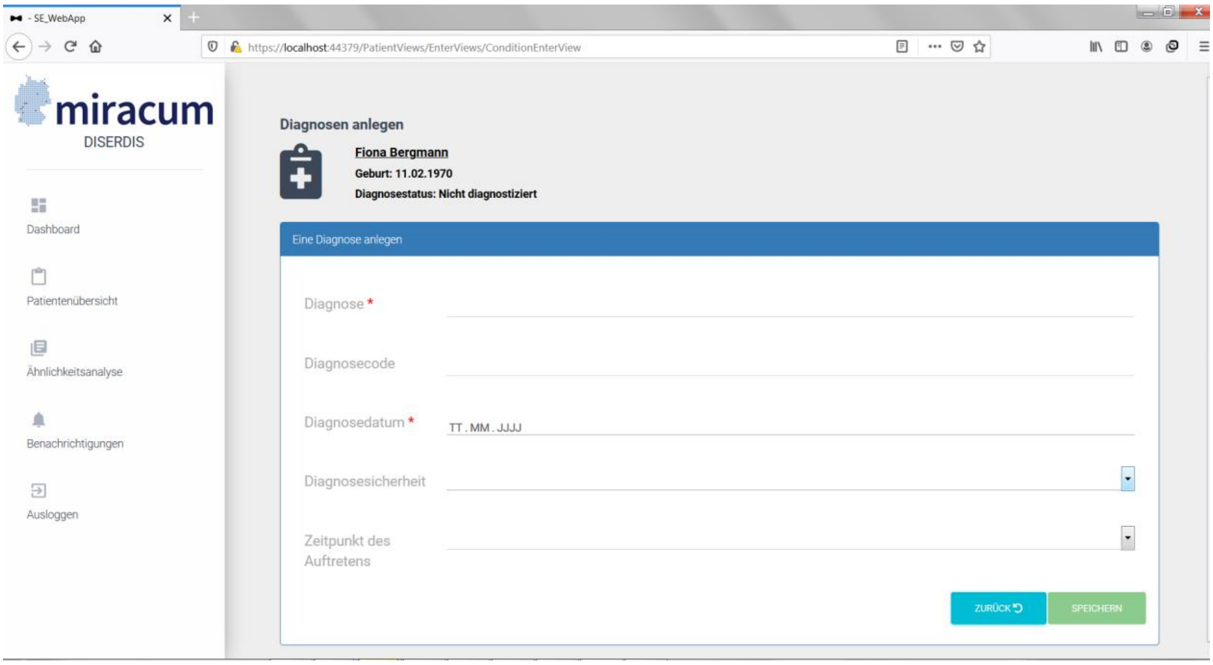

In addition to the symptom and diagnosis history view, the user can also create, delete or edit the family history, as shown in the next figure. This contains only one entry regarding the consanguinity between the parents of the patients.

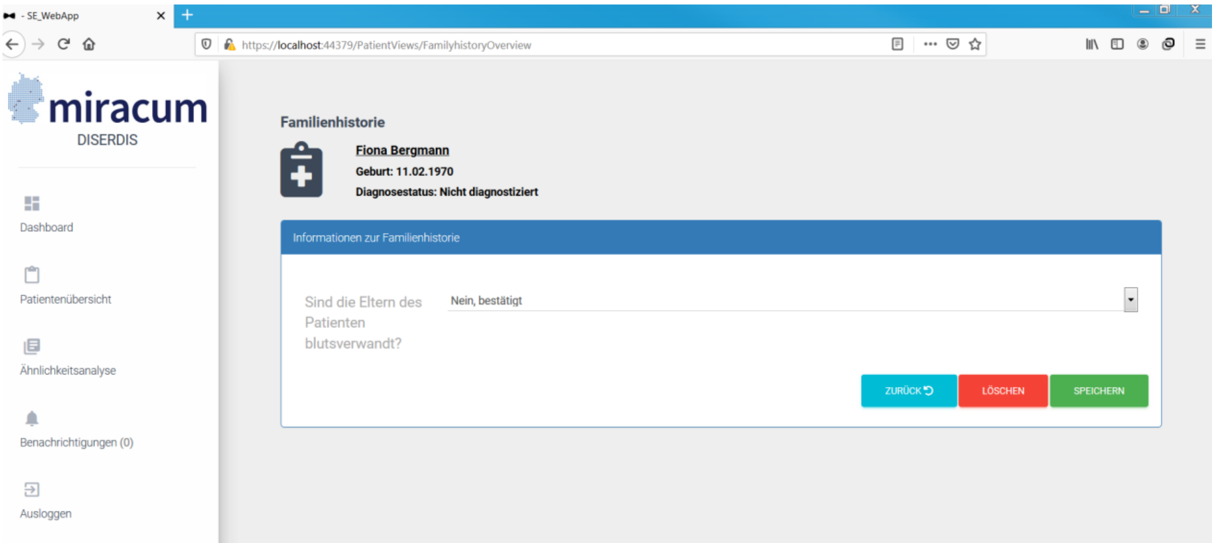

Execution of the similarity analysis

After explaining the menu structure and the management of patient data, the user interaction with the decision support function is shown. In order to use the decision support function with the similarity analysis, the user can click on the button “Similarity Analysis (Ähnlichkeitsanalyse)” in the left menu panel of the system. The existing locations, where the similarity analysis can be performed, are displayed. Here, the location (Standort), the availability of the location (Verfügbarkeit), a checkbox to select the location (Analyse durchführen) and the contact person (Ansprechpartner) at the location are displayed. To execute the similarity analysis, the button “Send request for similarity analysis (Anfrage zur Ähnlichkeitsanalyse versenden)” must be clicked to send a request to the locations.

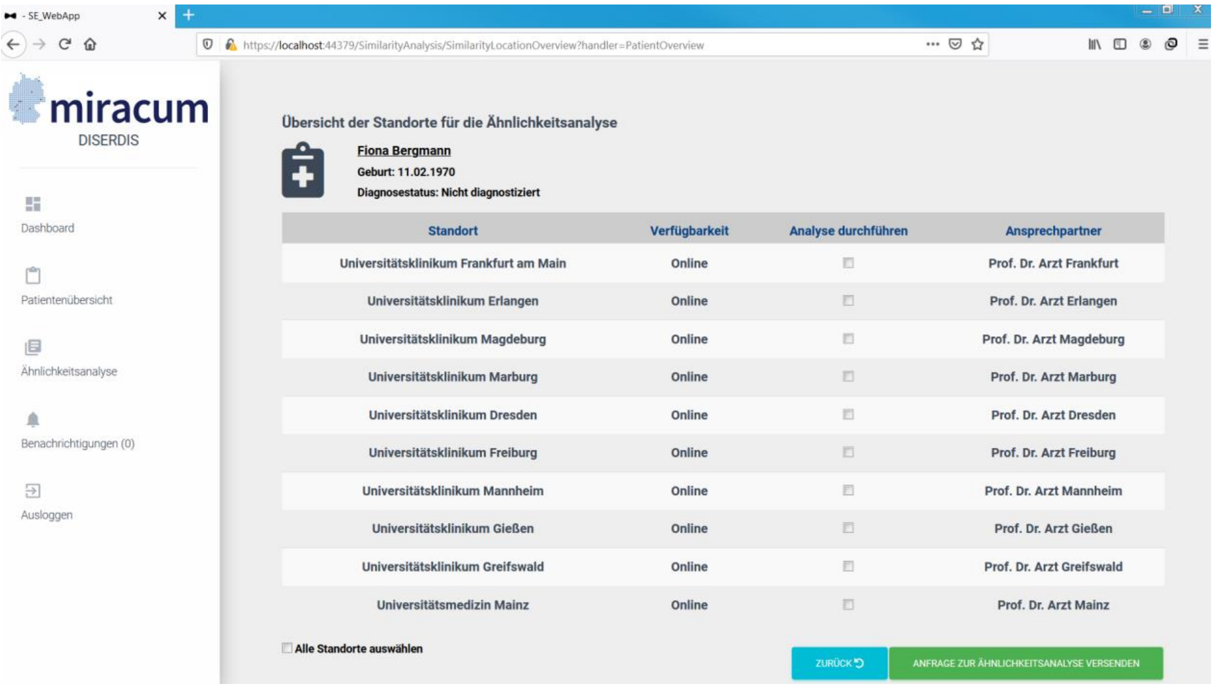

After the execution of the similarity analysis, the user will receive a message that the analysis is being carried out and the process may take some time (see figure below). Meanwhile, the user can continue working with the CDSS and will be notified via a notification, when the result is available.

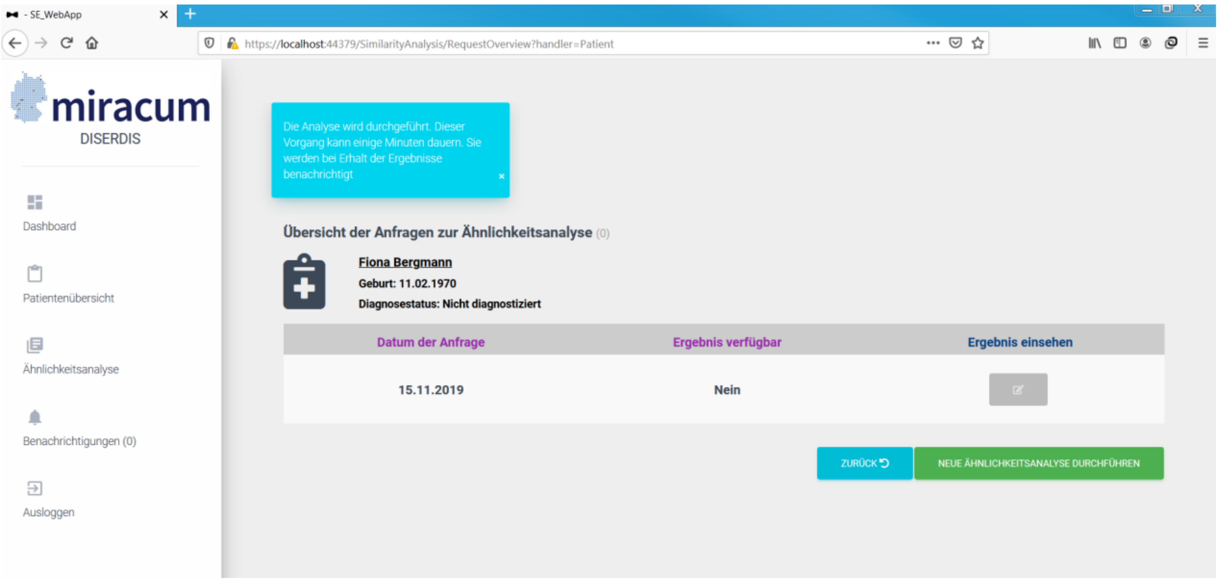

If the similarity analysis is successful, a green notification appears in the centre of the screen as shown in the next figure. The user is informed that the result of the similarity analysis is available. In addition, the number of notifications is incremented in the left menu "Notifications (Benachrichtigungen)" and highlighted in red (see figure below).

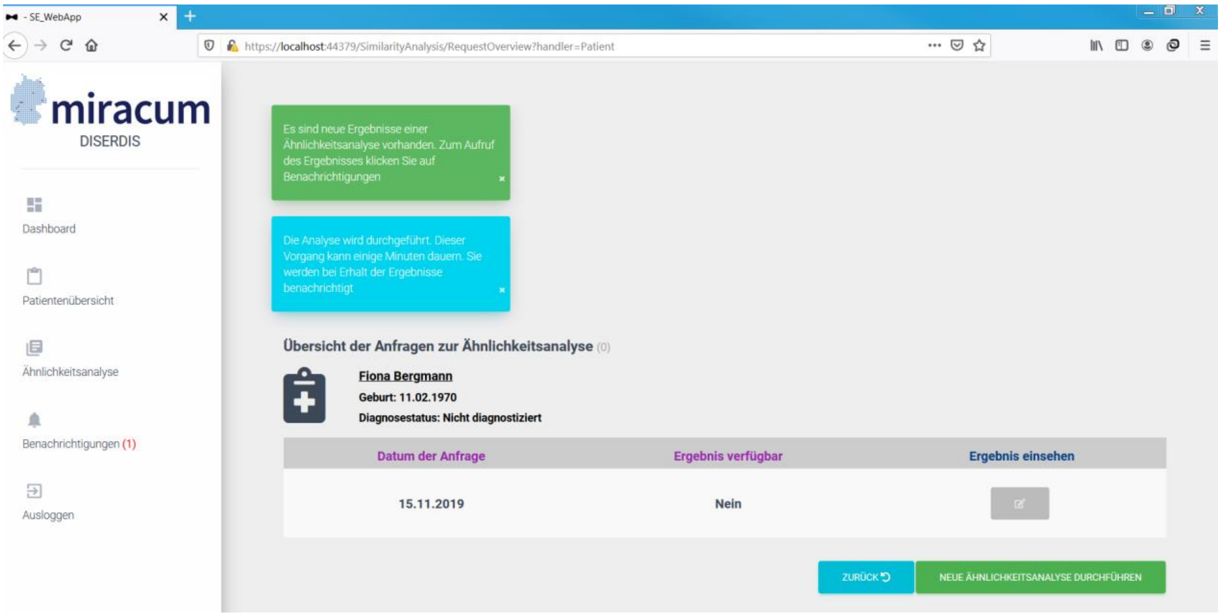

If the user opens this notification, an overview of the similarity analysis results is shown. As shown in the following figure, all analysis that have been executed with the CDSS are displayed. For each similarity analysis that has been performed, the name of the patient (Name) for whom the similarity analysis has been performed, date of birth (Geburtsdatum), date of request (Datum der Anfrage) and whether the result is already available (Ergebnis verfügbar) are displayed. The details of the similarity analysis can be viewed by clicking the button “View result (Ergebnis einsehen”).

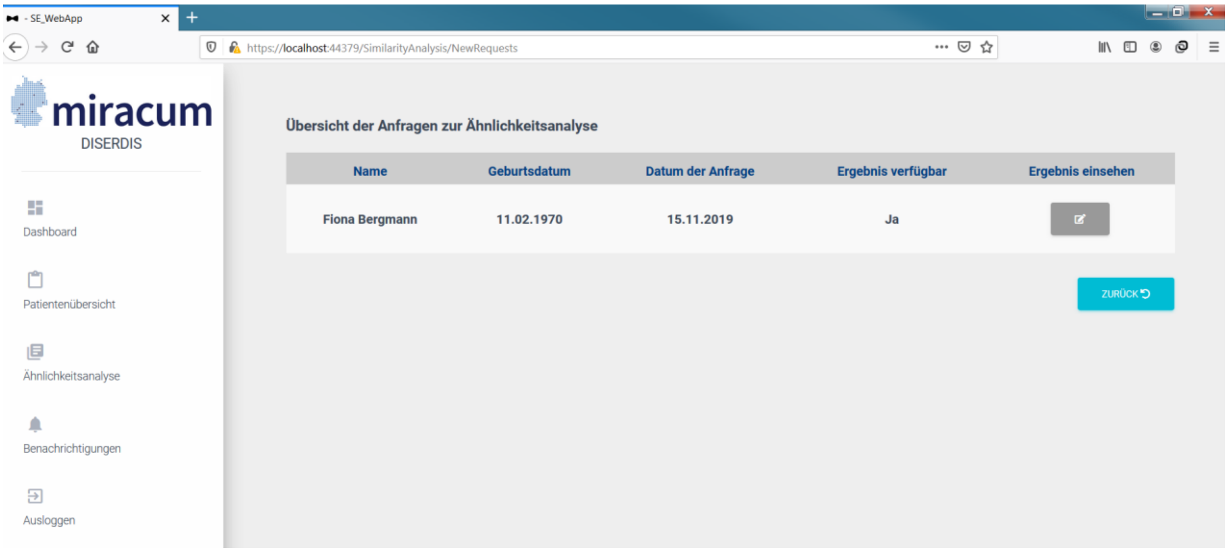

Results of the similarity analysis

The results of the similarity analysis are presented for the patient for whom the similarity analysis was performed. The user can switch between four panels to investigate the results of the similarity analysis. The following panels are available and shown in the figure below:

- Overview of similar patients (Ähnliche Patienten anzeigen (Übersicht))
- Patient comparison (Patienten vergleichen)
- Patient timeline (Patienten Historie)
- Medical history (Verlaufsparameter)

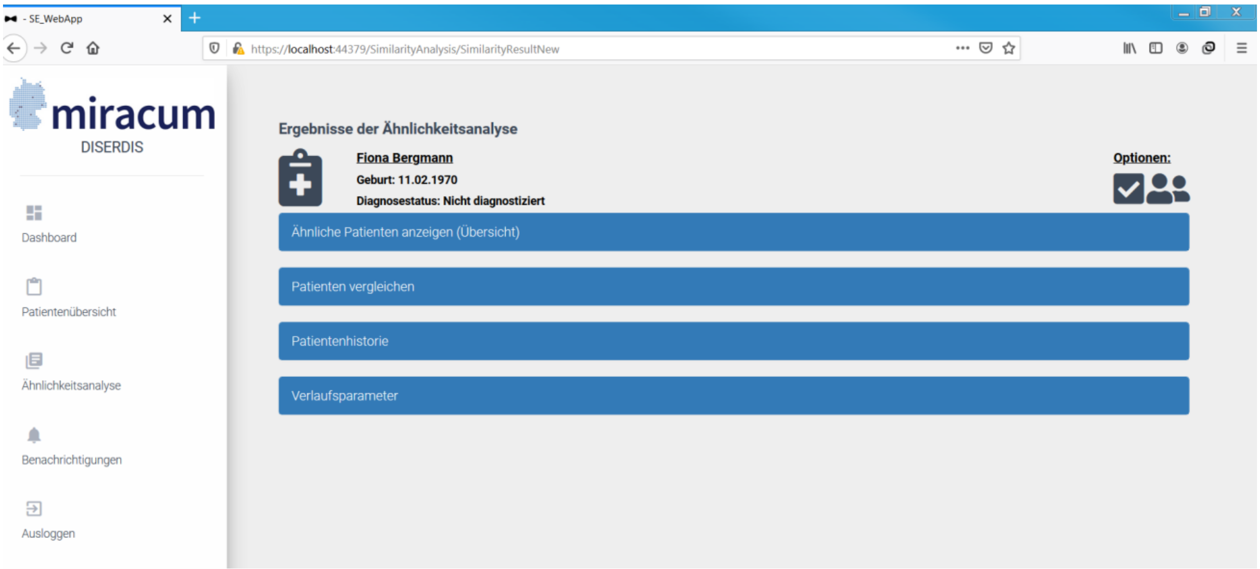

Overview of similar patients

In the "Panel Overview of similar patients (Ähnliche Patienten anzeigen (Übersicht))", a table with the similiar patients is shown (see figure below). In that table, the similarity is shown as a percentage (Ähnlichkeit (%)) and other information such as diagnosis (Diagnose), diagnosis code (Diagnosecode), classification of the diagnosis code (Klassifikation), location (Standort), date of birth (Geburtsdatum) and gender (Gender) of the patient are displayed. By clicking the "Select button (Auswählen)", the user can select the similar patient and view further details in the panels below.

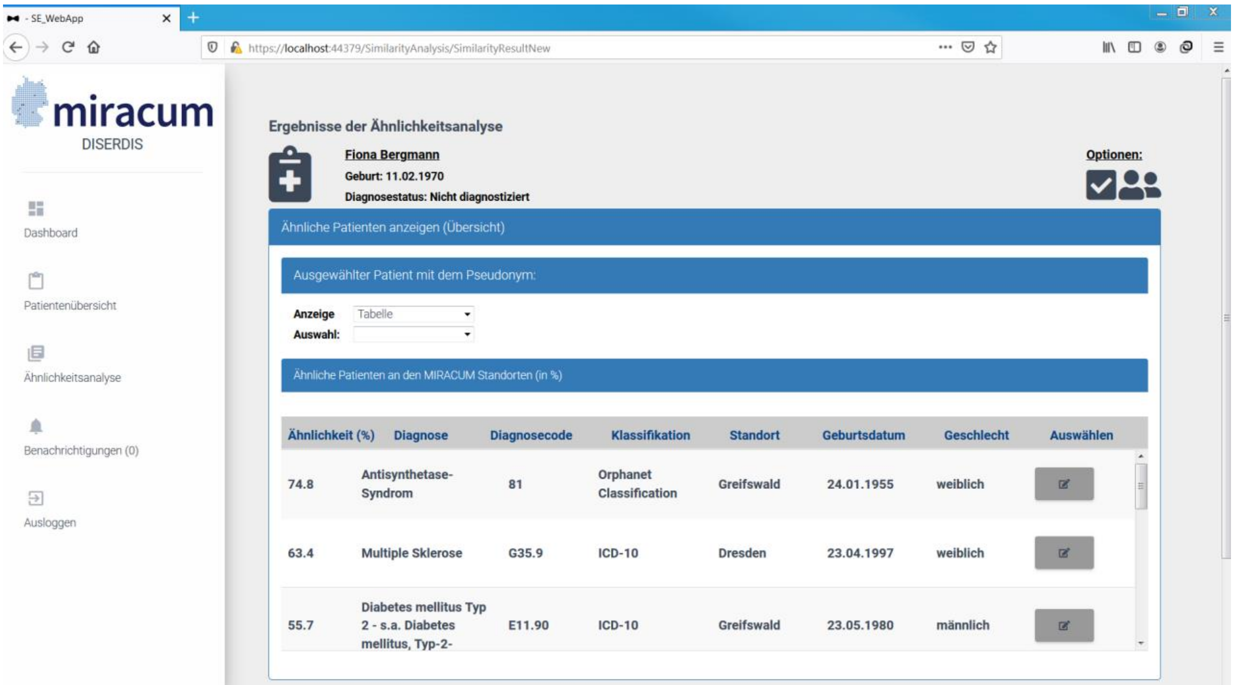

In the following figure a “patient similarity plot” as a scatterplot is visualised, whereas the patients are sorted in ascending order of similarity. Similar to the tabular representation, the similar patient can be selected. The “Display (Anzeige)” drop-down menu allows to select between the “table”, shown in the figure above, and the “scatterplot”.

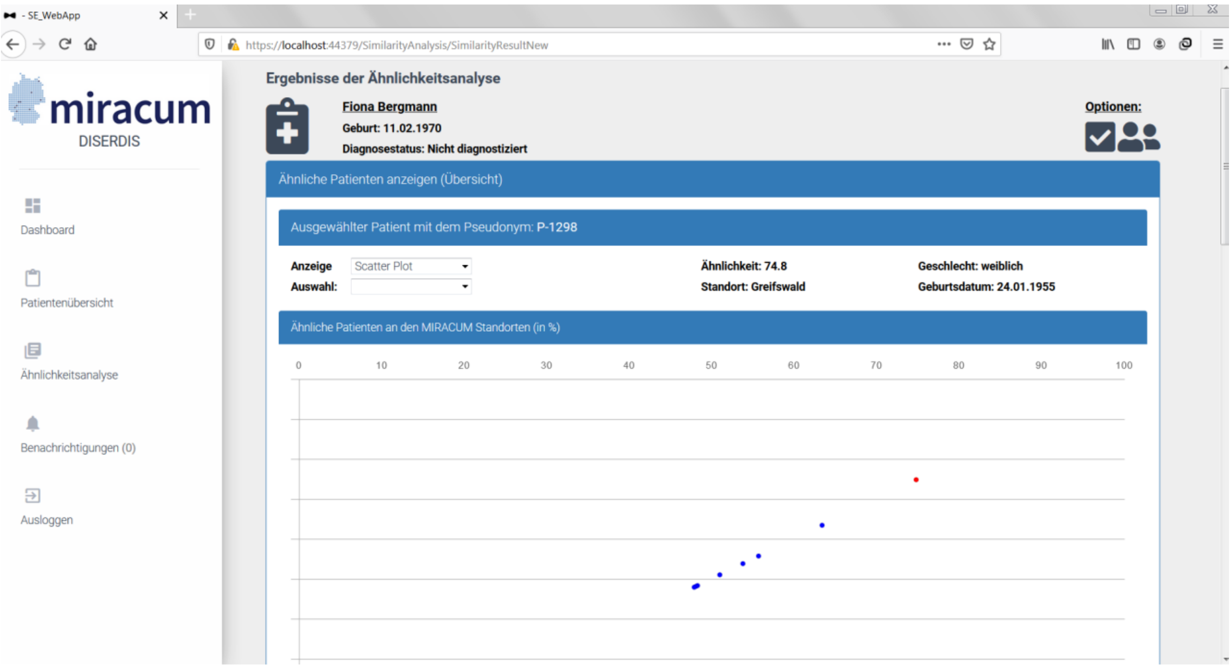

Patient comparison

The following figure shows the comparison of two patients (Patienten vergleichen). The undiagnosed patient, for whom the similarity analysis was performed, is compared to the selected similar patient. This provides the following information: diagnosis (Diagnose), rare disease (Seltene Erkrankung), family history (Familienhistorie), age at diagnosis (Alter bei Diagnose) and gender (Geschlecht).

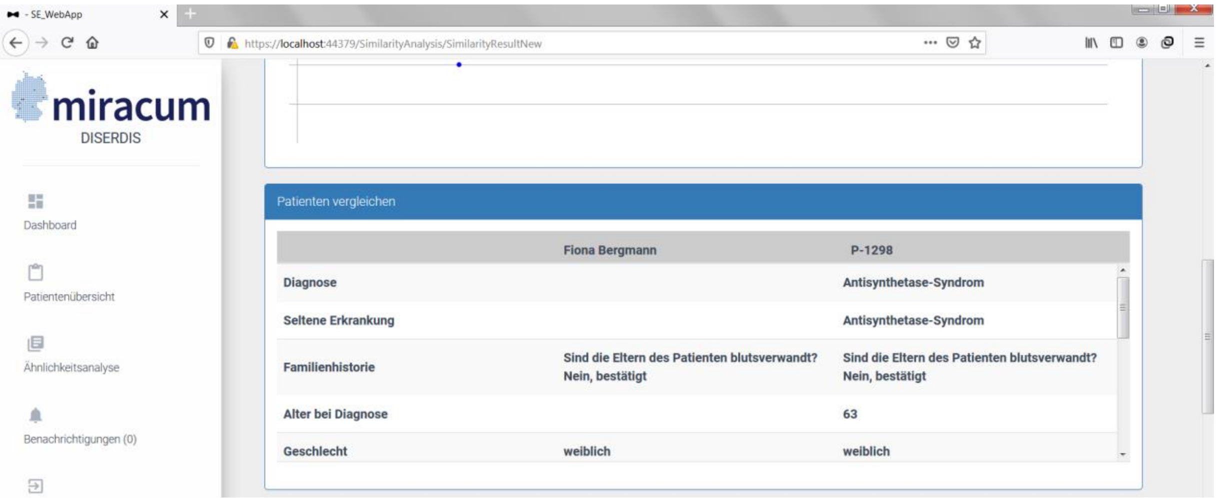

The following figure shows further information in the patient comparison. This includes the similarity between the two patients as well as the similarity of phenotypes (Ähnlichkeit der Phänotypen), diagnoses (Ähnlichkeit der Diagnosen), family history (Ähnlichkeit der Familienhistorie), age (Ähnlichkeit des Alters) and gender (Ähnlichkeit des Geschlechts).

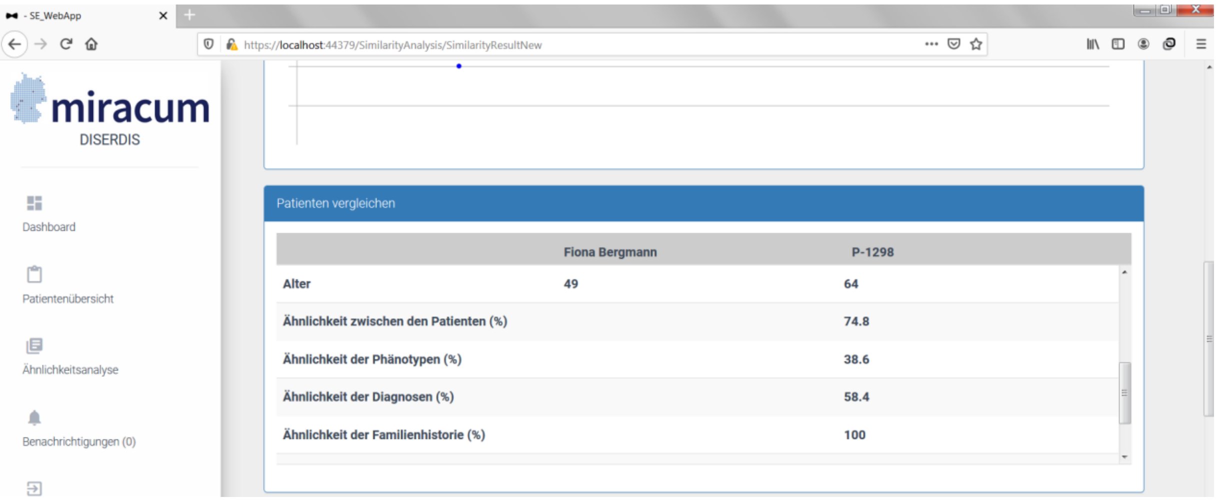

Patient timeline

The patient timeline (Patientenhistorie) is available as an additional panel, as shown in the following figure. Here, the diagnoses and symptoms of the similar patient are displayed over a time axis. By clicking on a timestamp, information on the diagnosis or the symptoms are shown.

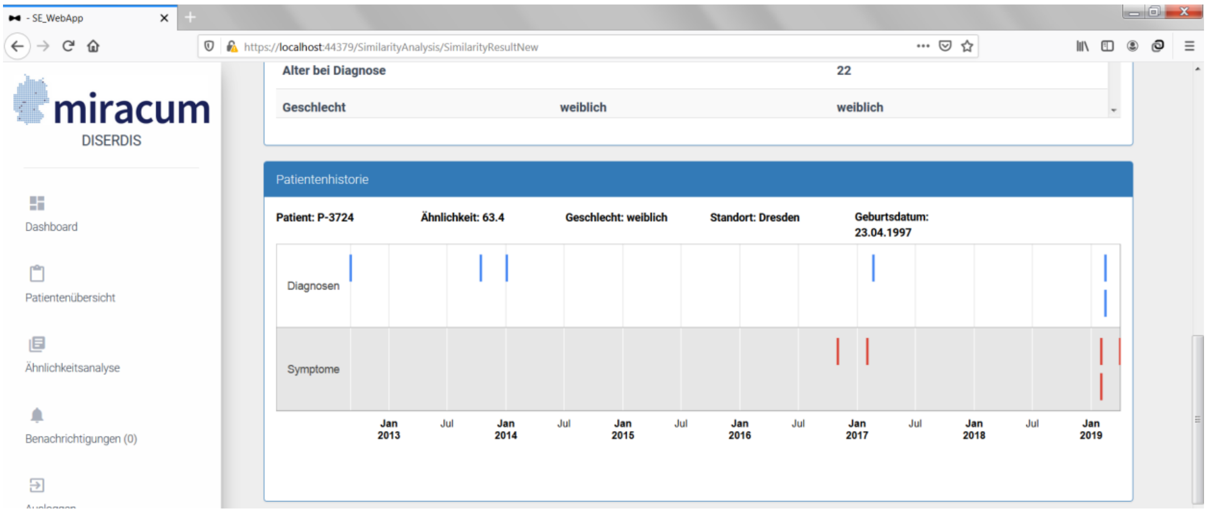

Medical History

The last panel is used to display observed medical parameters like blood pressure or lab values over the time. Here, parameters of two patients can be compared. Depending on which parameters are available for the patients, different parameters can be selected. The example in the following figure shows a comparison of blood pressure over the time.

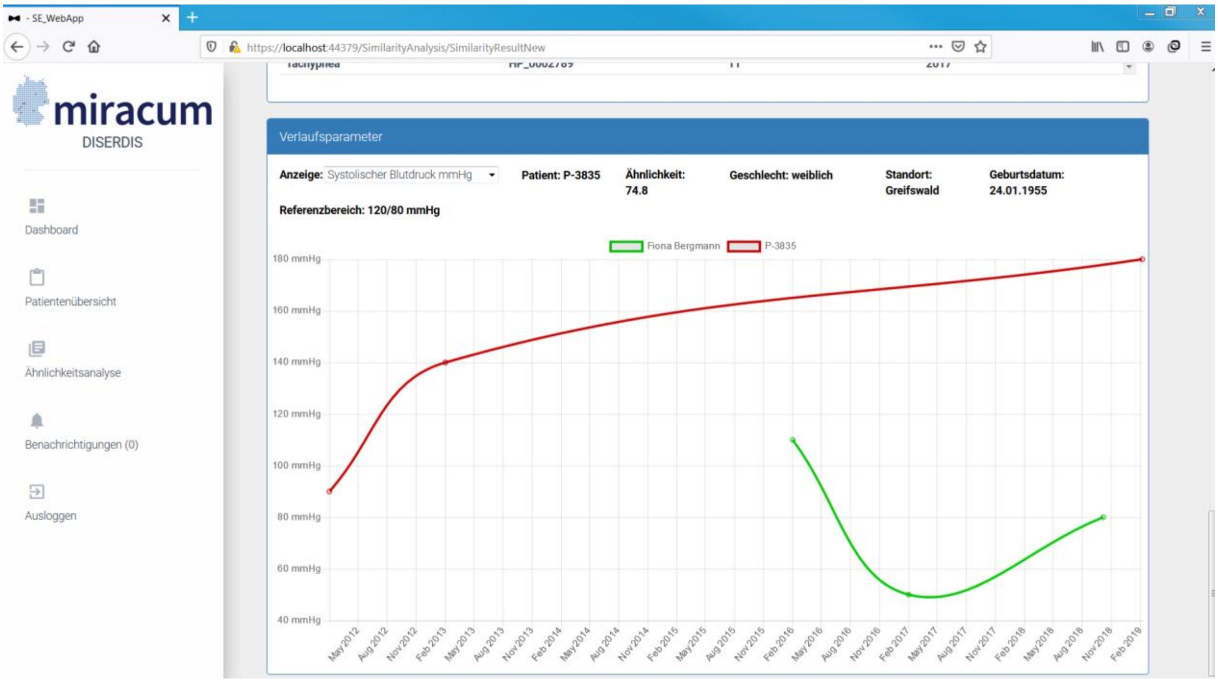

Criteria for similarity analysis

As shown in the following figure, two options can be selected in the view on the upper right side. On the one hand, it is possible to have the results of the similarity analysis calculated only on the basis of certain criteria. The following figure shows the selection of available criteria. By clicking on “Apply (Anwenden)”, the website is reloaded and all data of all panels are calculated based on the criterion only. The following criteria can be selected: diagnosis

(Diagnosen), symptoms (Symptome), age (Alter), gender (Geschlecht), Family History (Familienhistorie).

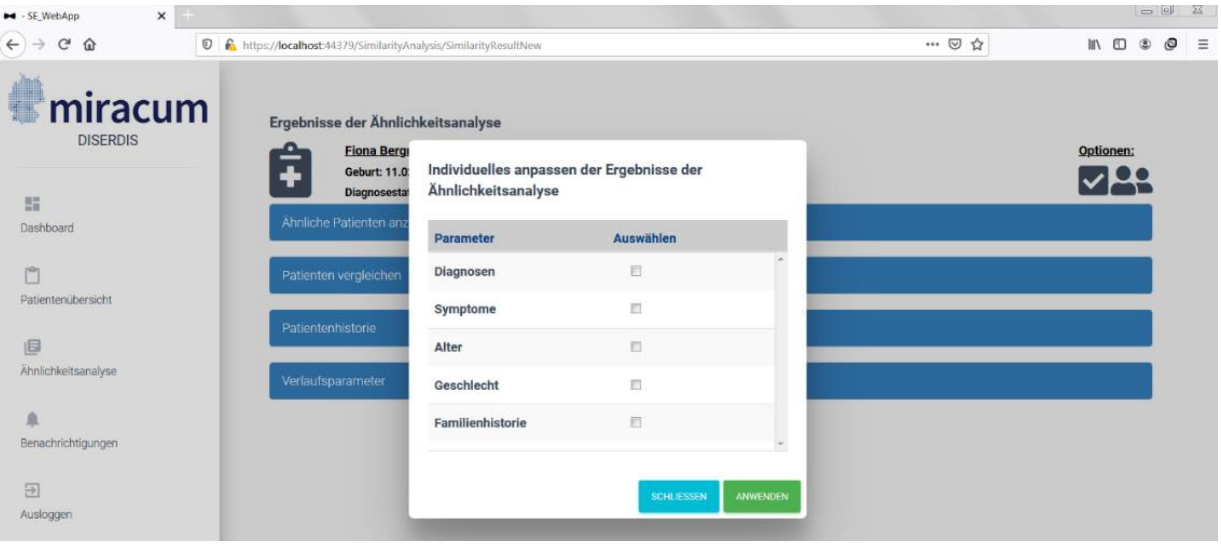

Search for experts

Another option is to search for a medical expert on a specific diagnosis. For this purpose an interface to the web portal "SE-Atlas" (www.se-atlas.de) was developed. The SE-Atlas provides a mapping of rare disease experts in Germany. The CDSS allows to select a patient with a diagnosis and then find the expert in the SE-Atlas without having to access the website. The following figure shows the results of the search for the diagnosis "Antisynthetase Syndrome". All experts found in the SE-Atlas with their contact information are shown.

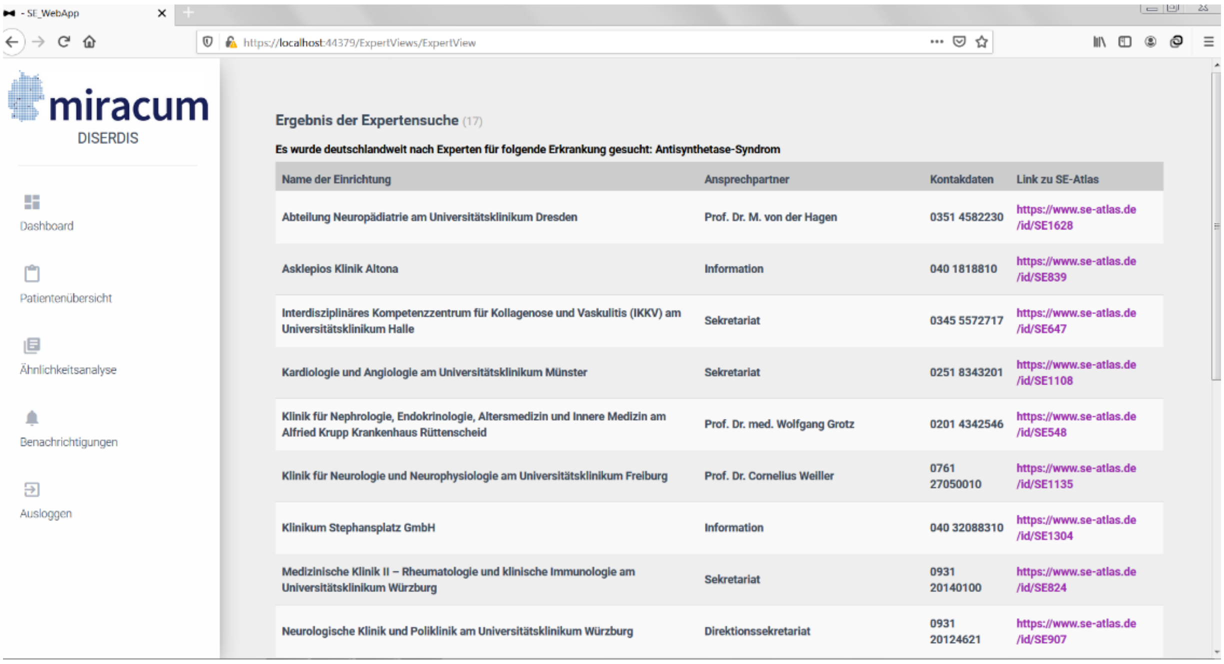

Supplement: Supplementary file 3 — Additional file 3. Functionality of the CDSS. [file 12911_2021_1435_MOESM3_ESM.pdf]
