# Supplementary material for: Evaluation of a clinical decision support system for rare diseases: a qualitative study
Source: BMC Med Inform Decis Mak. 2021 Feb 18;21:65. doi: 10.1186/s12911-021-01435-8 (PMC7890997; doi:10.1186/s12911-021-01435-8)
Supplement: Supplementary file 4 — Additional file 4. Instruction sheet. [file 12911_2021_1435_MOESM4_ESM.pdf]

Instruction sheet for Rare Diseases experts

Dear project partner,

Below you will find the instruction sheet for this study. You will use the diagnosis support system in order to get support in diagnosing a fictitious patient. You will find the patient in the system and then use the system to perform a similarity analysis for the patient.

The introduction sheet consists of three parts. Part A contains information about your patient. Part B shows the tasks to be performed in this study. In part C a questionnaire about the diagnosis support system has to be filled.

During the test procedure, you should tell what you think when you use the CDSS, which questions you have and what you do not understand. Any comments, both positive and negative, are very welcome.

Part A: Patient Information

The patient *Fiona Bergmann*, born on 1970, has been complaining of fever, dyspnoea and paraesthesia for several months. She also describes permanent fatigue and recurrent depressive episodes. In 2016, the patient was diagnosed with acute bronchitis, allergic asthma and upper respiratory tract infection.
The patient’s diagnoses and symptoms are shown below.

Previous diagnoses

The following diagnoses were documented about the patient. The diagnoses were given using the International Statistical Classification of Diseases and Related Health Problems (ICD-10).

Diagnosis	Diagnosis-Code (ICD-10)	Date of diagnosis	Diagnostic confidence	Time of occurrence
Acute upper respiratory infection, unspecified	J06.9	03.11.2016	Confirmed	Not clear
Predominantly allergic asthma	J45.0	15.12.2016	Confirmed	Not clear
Acute bronchiolitis, unspecified	J20.9	10.12.2016	Confirmed	Not clear

Symptoms

The following symptoms were documented about the patient. The symptoms were given using the Human Phenotype Ontology (HPO).

Symptom (Englisch)	Symptom-Code (HPO)	Month of first occurrence	Year of first occurrence
Dyspnea	HP:0002094	January	2019
Fatigue	HP:0012378	December	2018
Fever	HP:0032324	December	2018
Depressivity	HP:0000716	January	2019

Family History

Regarding the patient's family history, the following question was documented:

Question	Answer
Are the patient's parents blood relatives?	No confirmed

Part B: Tasks

Task 1:

Please find the patient *Fiona Bergmann* in the patient overview and get an overview of the patient details. Look at the symptom, diagnosis and family history. Please share your thoughts aloud during the assignment:

- What would you do next?
- What would you expect from the system?
- Where are problems and why?
- What is good, what is bad?

Task 2:

You have now obtained an overview of the patient's medical data. You can continue and perform out a similarity analysis with the system for the patient *Fiona Bergmann*. Please find the possibility in the system to perform a similarity analysis for the patient *Fiona Bergmann.* Please proceed as follows:

1. Find the possibility to perform a similarity analysis and select the patient *Fiona Bergmann*.
2. Select all locations.
3. Send the request for similarity analysis.
4. Wait for the results. This process may take a few minutes. When the results are available, you can continue with Task 3.

Please share your thoughts aloud during the assignment:

- What would you do next?
- What would you expect from the system?
- Where are problems and why?
- What is good, what is bad?

Task 3:

The results of the similarity analysis for the patient *Fiona Bergmann* are available now. You can now view the results. Please proceed as follows:

1. Find the possibility to view the results of the similarity analysis for the patient *Fiona Bergmann*.
2. Please check which is the most similar patient, what gender he/she is and when he/she was born. Afterwards, select the most similar patient.
3. Please check which diagnosis the patient has and which classification was used to document the diagnosis.
4. Please find the possibility to display the overview of similar patients as a scatterplot.

5. Please find the possibility to compare the patient *Fiona Bergmann* with the most similar patient. Then compare the displayed parameters.
6. Find the possibility to view the patient timeline of the most similar patient. Please check which diagnoses and symptoms were diagnosed. Also find the opportunity to view the diagnoses and symptoms in detail.
7. Find the possibility to view the medical history of the patient. Compare the patients' systolic blood pressure.
8. The system offers the possibility to filter the results of the similarity analysis according to certain criteria (e.g. a similarity comparison based on symptoms only). Please find the option in the system to have the similarities calculated on the basis of symptoms only.
9. Now, you want to find one or more experts to the rare disease of the similar patient. Please find the option in the system to list all experts to the rare disease.
Please share your thoughts aloud during the assignment:

- What would you do next?
- What would you expect from the system?
- Where are problems and why?
- What is good, what is bad?

Part C: Questionnaire

The final section of this study is designed to record your personal assessment of the diagnosis support system. Afterwards, some personal data will be required for evaluation. Like all data in this study, the following information you provide will be collected anonymously.

User interface design

No.	Question	strongly disagree	disagree	neutral	agree	strongly agree
1	I think that I would like to use this system frequently.	☐	☐	☐	☐	☐
2	I found the system unnecessarily complex.	☐	☐	☐	☐	☐
3	I thought the system was easy to use.	☐	☐	☐	☐	☐
4	I think that I would need the support of a technical person to be able to use this system.	☐	☐	☐	☐	☐

No.	Question	strongly disagree	disagree	neutral	agree	strongly agree
5	I found the various functions in this system were well integrated.	☐	☐	☐	☐	☐
6	I thought there was too much inconsistency in this system.	☐	☐	☐	☐	☐
7	I would imagine that most people would learn to use this system very quickly.	☐	☐	☐	☐	☐
8	I found the system very cumbersome to use.	☐	☐	☐	☐	☐
9	I felt very confident using the system.	☐	☐	☐	☐	☐
10	I needed to learn a lot of things before I could get going with this system.	☐	☐	☐	☐	☐

Overview of patient data

No.	Question	strongly disagree	disagree	neutral	agree	strongly agree
11	The system allows me to easily get an overview of all patients.	☐	☐	☐	☐	☐
12	The system allows me to easily get an overview of patient's symptoms.	☐	☐	☐	☐	☐
13	The system allows me to easily get an overview of patient's diagnosis.	☐	☐	☐	☐	☐
14	The system allows me to easily get an overview of the patient's family history.	☐	☐	☐	☐	☐

Similarity analysis with the diagnosis support system

No.	Question	strongly disagree	disagree	neutral	agree	strongly agree
15	It is easy to perform a similarity analysis for a patient.	☐	☐	☐	☐	☐

Presentation of the results of the similarity analysis

No.	Question	strongy disagree	disagree	neutral	agree	strongly agree
16	The diagnosis support system allows me easily to get an overview of the similar patients of the similarity analysis.	☐	☐	☐	☐	☐
16.1	Do you prefer the presentation of the results of the similarity analysis as scatterplot or table?	**Scatterplot** ☐			**Table** ☐	
16.2	I can easily determine which is the most similar patient, the diagnosis and gender of the patient and the location where the patient was treated.	☐	☐	☐	☐	☐
17	The patient comparison display allows me to easily compare data from two patients.	☐	☐	☐	☐	☐
17.1	I think the functionality of the patient comparison is relevant for diagnosis.	☐	☐	☐	☐	☐
18	The presentation of the patient' timeline allows me to easily get an overview of the patient's history.	☐	☐	☐	☐	☐
18.1	I think the presentation of the patient timeline is relevant for diagnosis.	☐	☐	☐	☐	☐
19	I think the presentation of the patient's medical history is relevant for diagnosis.	☐	☐	☐	☐	☐

No.	Question	strongy disagree	disagree	neutral	agree	strongly agree
20	To set criteria for similarity analysis is relevant for diagnosis.	☐	☐	☐	☐	☐
21	The diagnosis support system allows me easily to find an expert for a rare disease.	☐	☐	☐	☐	☐

Questions about yourself

No.	Question	Answer				
22	Gender	Male ☐		Female ☐		
23	Age group	20-29 ☐	30-39 ☐	40-49 ☐	50-59 ☐	>59 ☐
24	In which medical specialization do you work (e.g. neurology, pediatrics)?					
25	How many years' experience do you have in rare diseases?					
26	Do you have previous experience with diagnosis support systems?	Yes ☐		No ☐		
