# Supplementary material for: Evaluation of a clinical decision support system for rare diseases: a qualitative study
Source: BMC Med Inform Decis Mak. 2021 Feb 18;21:65. doi: 10.1186/s12911-021-01435-8 (PMC7890997; doi:10.1186/s12911-021-01435-8)
Supplement: Supplementary file 5 — Additional file 5. Example quotations of study participants. [file 12911_2021_1435_MOESM5_ESM.pdf]

Example quotations from the thinking aloud test

Note: Breakes in the pronunciation of the study participants are represented by (...)

Quotation number	Quotation (translated into English)	Quotation (original German)	Participant Number
1	"Well, everything I expected was there."	„Gut, alles was ich erwartet habe war da."	P1
2	"That means (...). I would now want more or less accurate doctor's letters or more detailed information (...). Because if I have to consider the patient as a rare disease case, then my work schedule would say, okay, rule out that it cannot be anything else frequent and then it would be (...). I would be most concerned with this fever, and (...) I would want to see exactly what has been done."	„Das heißt ich würde jetzt (…). Ich würde jetzt quasi genaue Arztbriefe wollen oder genauere Informationen (…). Weil wenn ich den Patienten jetzt als Fall von Seltenen Erkrankungen betrachten soll, dann würde meine Arbeitsschiene jetzt sagen, okay schließ aus, das es nicht irgendwas anderes Häufiges sein kann und dann wäre jetzt (…). Da würde mich jetzt am meisten beschäftigen, wäre jetzt irgendwie dieses Fieber, und (…) da würde ich jetzt quasi genau sehen wollen was genau gemacht worden ist."	P2
3	"But I have to say now, when I see this and I have the feeling that these are symptoms of a common disease, I don't realise that it says edit. What should I edit if I have not even been able to read the findings. I do not find it easy. I should be able to look at it critically now, because I don't know who created these findings. Whether they are correct or not. Or anyone else's. I have no possibility to check the quality of the findings."	„Aber ich muss jetzt sagen, wenn ich das hier sehe und ich habe das Gefühl, das sind Symptome von einer häufigen Erkrankung, dann ist mir nicht klar, hier steht editieren. Was soll ich editieren, wenn ich nicht einmal die Befunde habe lesen können. Ich finde die jetzt hier nicht leicht. Ich müsste das jetzt alles kritisch anschauen können, weil ich nicht weiß wer jetzt diese Angaben hier gemacht hat. Ob die korrekt sind oder eben noch nicht korrekt sind. Oder von irgendjemanden. Ich habe keine Möglichkeit die Qualität der Behauptungen zu überprüfen."	P3

Quotation number	Quotation (translated into English)	Quotation (original German)	Participant Number
4	“Right. So you gotta wonder where the symptoms come from. That would be good, of course, such a hyperlink link that I can say dyspnoea is documented, where do they come from. For example, if you click on it and see the findings, or if several people are working on the same file, then you would ask who documented these symptoms? This is of course different if the paraesthesia has been documented by a neurologist than by a psychiatrist or even an internist I am still missing some descriptive attributes here.”	„Genau. Also man wird sich ja fragen woher kommen die Symptome. Das wäre natürlich gut, so eine Hyperlink Verlinkung, dass ich sagen kann Dyspnoe ist dokumentiert, woher kommen die. Man klickt dann zum Beispiel drauf und kommt dann auf den Befund, oder wenn zum Beispiel mehrere Leute in einer Akte arbeiten, dann wer hat diese Symptome dokumentiert. Das ist natürlich was anderes, wenn die Parästhesie von einem Neurologen dokumentiert wurde als von einem Psychiater oder gar von einem Internisten, kann man auch schon sagen. Mir fehlen hier noch einige beschreibenden Attribute.“	P5
5	“So we don't see the patient, we see paper. And, no, that's not enough for me at all, because most diseases here are common diseases.”	„Also wir sehen ja den Patienten nicht, wir sehen Papier. Und, nein das reicht mir überhaupt nicht, da hier die meisten Erkrankungen häufige Erkrankungen sind.“	P3
6	“So, basically, it is not enough for me. But it depends on whether this is a first, what are the symptoms. Then of course it is sufficient. But in principle I would like to have details. In the case of dyspnoea in particular, this is high dyspnoea, dyspnoea caused by stress, and in case of fever, how high are the temperatures really, is this a chronic increased body temperature or fluctuating symptoms. The same applies to paraesthesia, i.e. which extremities are affected. Paraesthesia can have	„Also, grundsätzlich reicht es mir nicht. Es ist aber, hängt also sozusagen davon ab, ob das ein erster, eine erste Übersicht sein soll, was sind das für Symptome? Dann ist es natürlich ausreichend. Aber vom Prinzip her wünsche ich mir Details. Gerade zur Dyspnoe, ist das eine hohe Dyspnoe, eine Belastungsdyspnoe, beim Fieber, wie hoch sind denn wirklich die Temperaturen. Ist das eine chronische erhöhte Körpertemperatur oder Schwanken Symptomatik. Bei den Parästhesien genauso,	P6

Quotation number	Quotation (translated into English)	Quotation (original German)	Participant Number
	very different qualities. Depressiveness would suffice and fatigue, which are more descriptive. But this is not enough for me. But to have a first overview, then yes."	also welche Extremitäten sind Betroffen. Parästhesien können ja sehr unterschiedliche Qualitäten haben. Gut, Depressivität würde ausreichen und Fatigue, das sind ja eher so beschreibende. Aber bei den ersten Drei würde mir das Prinzipiell nicht ausreichen. Aber wenn es darum geht einen ersten Überblick zu haben, dann ja."	
7	"So, this is just the way I would code it, if I want to code the case. But if I don't see the patient now, which is our classic case in the A-centre, then I have to know what kind of dyspnea it is. Is that dyspnea when she's run a mile."	„Also das ist jetzt halt so wie ich's kodieren würde, wenn ich den Fall kodieren will. Aber wenn ich jetzt die Patientin dazu nicht sehe, was jetzt im A-Zentrum unser klassischer Fall ist, dann muss ich wissen was ist das für eine Dyspnoe. Ist das eine Dyspnoe, wenn sie einen Kilometer gelaufen ist."	P2
8	"I'll check it out. Wait a minute. Dyspnea, fever, paresthesia. Well, with paresthesia it would be very gross, of course. Of course you would like to know what is meant more precisely. Because it is a generic term. And it does not include whether it is on the legs, on the feet, on the arms, on the hands, on one side or both sides. And then, of course, there are differences. Paresthesia only means that the sensation is changed, but not in what way."	„Ich guck mal. Warte mal. Dyspnoe, Fieber, Parästhesien. Ja also, bei den Parästhesien wäre es natürlich sehr grob. Da würde man natürlich schon gerne wissen, was damit genauer gemeint ist. Weil das ein Oberbegriff ist. Und es jetzt nicht beinhaltet, ob es an den Beinen, an den Füßen, an den Armen, an den Händen, einseitig, beidseitig. Und dann gibt es natürlich noch Unterschiede. Parästhesien bedeutet ja nur, dass das Empfinden verändert ist, aber nicht in welcher Weise."	P7
9	"Right. That would not be enough for me. The family anamnesis then includes, independently of asking about diseases, depending on the	„Genau. Das wäre mir nicht ausreichend. Also zur Familienanamnese gehören dann, unabhängig davon nach Erkrankungen zu fragen, je	P6

Quotation number	Quotation (translated into English)	Quotation (original German)	Participant Number
	age of the patient, what other relatives are there in your direction. Does the patient have children, does she have siblings, are the parents still alive? And then there are diseases that are interesting in both directions. For example oncological diseases, haematological diseases. I always ask if the children are healthy, depending on their age. Whether there are problems, whether there have been deaths before. Whether they have anything special to report. So that would not be enough for me."	nach Alter des Patienten, was gibt es in deiner Richtung noch für Verwandtschaft. Hat die Patientin Kinder, hat sie Geschwister, leben die Eltern noch? Und dann gibt es in beide Richtungen Erkrankungen die interessant sind. Zum Beispiel onkologische Erkrankungen, hämatologische Erkrankungen. Ich frage immer nach, je nachdem, ob es sich aus dem Alter ergibt, ob die Kinder gesund sind. Ob es Probleme gibt, ob es vorher Todesfälle gibt. Ob Sie irgendetwas besonders zu berichten hat. Also das würde mir nicht reichen."	
10	"Other family members affected (...). For example, and whether these diagnoses occur in other family members."	„Andere Familienmitglieder betroffen (…). Zum Beispiel und ob diese Diagnosen bei anderen Familienmitgliedern auftreten."	P3
11	"Yes, okay. So, basically, it's clearly consanguinity. It's not common. And of course, well that are of course all very unspecific symptoms. But if you now have a relatively specific disease, then I would of course be interested to know if it exists in your parents or close relatives, which is very similar. Daughter has cysts, kidneys, someone of the parents, maternal or paternal or relatives, they also have such a disease. That would actually interest me more than consanguinity."	„Ja gut. Also. Im Prinzip klar Blutsverwandtschaft. Kommt nicht so häufig vor. Und was natürlich, gut das natürlich auch alles ganz unspezifische Symptome. Aber wenn Sie jetzt eine relativ spezifische Krankheit haben, dann würde ich mich natürlich interessieren, gibt es das bei den Eltern oder bei der näheren Verwandtschaft, was sehr ähnlich ist. Tochter hat Zysten, Nieren, jemand von den Eltern, mütterlichen oder väterlichen oder Verwandten, haben die auch so eine Krankheit. Also das würde mich jetzt eigentlich mehr interessieren, als die Blutsverwandtschaft."	P4

Quotation number	Quotation (translated into English)	Quotation (original German)	Participant Number
12	“Yeah, well. Of course, right. I would expect more. Almost a kind of a family tree of the patients. And especially in the context of rare diseases, family members have similar symptoms. Which would be great for such a system, if maybe the family tree would appear. Then at I click on the brother and see (…). And I actually see the same patient overview that I now have for Ms Bergmann, then just for Lars Bergmann or for the brother, for example. That would be quite interesting. Yes of course, consanguinity is a marker, but not for all diseases.”	„Ja also. Natürlich klar, also. Man würde hier natürlich mehr erwarten. Fast schon eine Art Stammbaum der Patienten. Und insbesondere im Kontext Seltene Erkrankungen, haben die Familienmitglieder ähnliche Symptome. Was ja toll wäre bei so einem System, wenn jetzt vielleicht der Stammbaum auftauchen würde. Dann kommt irgendwann der Bruder, ich klick auf den Bruder und sehe (…). Und sehe eigentlich dieselbe Patientenübersicht, die ich jetzt für Frau Bergmann habe, dann eben für Lars Bergmann oder für den Bruder zum Beispiel. Das wäre ja ganz interessant. Ja natürlich, die Blutsverwandtschaft ist ein Marker sozusagen, aber auch nicht für alle Erkrankungen.“	P5
13	“In that in the constellation as it exists right now, this is not my first question. Nevertheless, this is interesting, because whenever you have to make a diagnosis, it is not uninteresting to investigate consanguinity. Because paediatrics 80% are genetic. That is super interesting. But in the symptom complexes that were given here, that would not have been my first question.”	„Sagen wir mal so, in der Konstellation wie sie jetzt gerade besteht, ist das nicht meine erste Frage. Das ist trotzdem interessant, weil wann immer man, wenn es um Diagnosen geht, das nicht uninteressant ist, wenn es Blutverwandtschaft geht. Denn gerade jetzt in der Kinderheilkunde sind 80 % genetisch sind. Das ist super interessant. Aber jetzt in den Symptomen, in den Symptomkomplexen, die hier angegeben wurden, wäre das jetzt nicht meine erste Frage gewesen.“	P7
14	"It is easy to use. No problem at all. ”	„Lässt sich einfach bedienen. Gar kein Problem.“	P1

Quotation number	Quotation (translated into English)	Quotation (original German)	Participant Number
15	"That was easy for me to find now because we work with a similar system."	„Das war jetzt leicht für mich zu finden, weil wir ja auch mit einer Telemedizinakte arbeiten. Die ähnlich ist.“	P6
16	“Then we go to the patient overview, that is still easy (...). And then it's the top one, Fiona Bergmann.”	„Dann gehen wir auf die Patientenübersicht, das ist ja noch einfach (…). Und dann ist es die oberste, die Fiona Bergmann.“	P4
17	“But otherwise, it is clearly evident. Are the patients, are the patient's parents blood relatives. Exactly.”	„Aber sonst, ist es klar ersichtlich. Sind die Patienten, sind die Eltern des Patienten blutverwandt. Genau.“	P5
18	“Otherwise, the mask is clearly arranged.”	„Ansonsten von der Maske her ist es übersichtlich.“	P5
19	"The age would be relevant, automatically calculated. The age of the patient when the symptoms occur. Up here is the date of birth. What I would like to see here in the overview is at least the sex and the automatic calculation of the age at the current time. That is something that the most systems offer.”	„Das Alter wäre, das wäre relevant sozusagen automatisch schon berechnet. Das Alter des Patienten bei Auftreten der Symptome. Was ich mir wünschen würde, wäre, hier noch in der Übersicht zumindest das Geschlecht und die automatische Berechnung des Alters zum aktuellen Zeitpunkt. Das ist ja etwas, was die generellen Akten ja auch immer bieten.“	P5
20	"And behind each one of these diagnoses, there needs a checkbox, okay, that's not it. And that's what the young colleagues have to learn, that they really check these kinds of diagnoses, which are sometimes made for accounting reasons. So they have to check the diagnosis according to medical guidelines.”	„Und hinter jeder dieser Diagnosen würde sich für mich so ein Checkhaken befinden müssen, okay, das ist es nicht. Und das ist das was die jungen Kollegen eben auch lernen müssen, dass sie zu dieser Art von Diagnosen, die ja manchmal auch aus Abrechnungstechnischen Gründen erstellt worden sind, wirklich checken, habe ich aus dem was auf dem Papier vorliegt, das leitliniengerecht	P2

Quotation number	Quotation (translated into English)	Quotation (original German)	Participant Number
		geprüft. Also das müssen sie dann nochmal leitliniengerecht prüfen.“	
21	“It's a bit hidden. This belongs further up. Because, here it is still possible now, because the list stops before that. If the list is longer, you won't see the button.”	„Das ist ein bisschen versteckt. Das gehört weiter nach oben. Weil, hier geht es jetzt noch, weil die Liste vorher aufhört. Wenn die Liste länger ist, siehst du den Knopf nicht mehr.“	P1
22	"Okay. It would be great if you put that up there. Because it's just totally unintuitive when I'm looking for the bottom left.”	„Okay. Da wäre es natürlich toll, wenn man das da oben platziert. Weil das ist halt total unintuitiv, wenn ich das da unten links suche“.	P7
23	“Send the request. Send (...). The operation may take a few minutes. (…) What would you expect from this system? Perhaps a nice message. There is a message. Okay, first the hourglass and then a message (...). Here I have a problem. How long has the window been open? Is my computer crashed?”	„Senden Sie die Anfrage ab. Versenden (…). Der Vorgang kann einige Minuten Zeit in Anspruch nehmen. (…) Was würden Sie vom System erwarten. Vielleicht eine nette Meldung. Jetzt kommt die Meldung. Okay, erst die Sanduhr und dann kommt doch noch eine Meldung (…). Hier hätte ich so ein bisschen das Problem. Wie lange steht das Fenster? Ist mein Computer abgestürzt?“	P1
24	“But I think that's well done. That the user is always informed about what is happening at the moment (...). Well, that didn't take that long, 45 seconds or so. 30 maybe even.”	„Das finde ich aber gut gemacht. Das der User immer über so ein Gif oder sowas informiert ist, was gerade passiert (…). Gut, das hat jetzt gar nicht so lange gedauert, 45 Sekunden oder so. 30 vielleicht sogar.“	P5
25	“For us the date of birth would be irrelevant.”	„Für uns wäre das Geburtsdatum unerheblich.“	P1

Quotation number	Quotation (translated into English)	Quotation (original German)	Participant Number
26	“The gender, yes okay (...). Rather the age at diagnosis. Because if I have an ancient case in there now and I'm ten years ahead. It's a classic finding that always occurs at 30. Then I'm 10 years ahead, then the patient here is 40. Then I definitely don't think that this is similar to mine (...). Because the date of birth has no relevance here.”	„Das Geschlecht, ja okay (…). Eher das Alter bei Diagnosestellung. Weil, wenn ich jetzt einen uralten Fall drin habe und ich bin 10 Jahre weiter. Es geht um einen klassischen Befund, der mit 30 immer auftritt. Dann bin ich 10 Jahre weiter, dann ist der Patient hier 40. Dann komm ich ja unbedingt nicht drauf, dass der ähnlich zu meinem ist (…). Denn das Geburtsdatum hat hier keine Relevanz.“	P1
27	“Here again: Year of the first manifestation. Is exactly the same as with the date of birth. That is actually (...). Irrelevant. Therefore rather age at first manifestation. Month and year.”	„Auch hier wieder: Jahr des ersten Auftretens. Ist genau das Gleiche wie mit dem Geburtsdatum. Das ist ja eigentlich (…). Irrelevant. Daher e-her Alter beim ersten Auftreten. Also Monat und Jahr (…).“	P1
28	“Yes again, the date is not interest. The age is interesting.”	„Ja auch hier wieder, das Datum interessiert mich nicht. Hier interessiert das Alter.“	P1
29	“I think ten years ahead. You got a 90-year-old patient. He’s dead now. Who cares if he died? I just want the age at onset.”	„Ich denk jetzt 10 Jahre weiter. Du hast jetzt einen 90-Jährigen Patienten drin. Der ist jetzt gestorben. Ja ist doch jetzt egal, ob der jetzt Tod war. Ich will ja nur wissen in welchem Alter hatte er das.“	P1
30	“Now I'm curious, let's just take age and gender (...). Now the system is being tested (...). Okay, now I have the problem again, because I have compared by age, the age should be show. That is what I already told. That is very important. And age is also (...). It should be "age at diagnosis.”	„Jetzt bin ich mal gespannt, jetzt nehmen wir mal nur Alter und Geschlecht (…). Jetzt wird das System getestet (…). Okay, jetzt habe ich wieder das Problem, weil ich nach Alter verglichen habe, müsste hier jetzt auch das Alter stehen. Das ist ja das was wir eben schon hatte. Das ist ganz wichtig. Und Alter ist ja auch	P1

Quotation number	Quotation (translated into English)	Quotation (original German)	Participant Number
		(…). Es müsste heißen Alter bei Diagnosestellung.“	
31	“That is interesting, of course. Is it possible to select other parameters here?”	„Das ist natürlich interessant. Kann man hier auch andere Parameter auswählen?“	P6
32	“So generally it is good. No question about it. It would just be super unfavourable if some poor assistant had to type it in by 10 in the evening.”	„Also generell ist es gut. Keine Frage. Es wäre jetzt nur super ungünstig, wenn das irgendein armer Assistenzarzt bis Abends um 10 eintippen muss.“	P7
33	“Right. So timeline is well. But all right, you can go like this. The measuring points could be highlighted a little more clearly. Otherwise, I think it's all about the general concept.”	„Genau. Also Zeitlinie ist gut. Klar ist immer die Frage sozusagen, wie mittel ich sozusagen. Also welche Linie. Also hier ja genau. Hier ist ja es ja smooth sage ich mal. Ja klar. Ist immer so die Frage, natürlich. Aber gut, kann man gerne so machen. Die Messpunkte noch etwas deutlicher hervorgehoben. Sonst ist generell ich glaube es geht ja um das generelle Konzept.“	P5
34	“So what you want to compare. Of course you want to compare parameters over time. That is certainly very important. So measurement parameters. Exactly. Of course, one must also consider converting units. And so on. But the database alone should provide that. But otherwise it's accurate. I like it.”	„Also, was man vergleichen will. Klar man will Parameter vergleichen über die Zeit. Das ist sicherlich sehr wichtig. Also Messparameter. Genau. Muss man natürlich bedenken auch, Einheiten umrechnen. Und sowas. Aber das sollte ja allein die Datenbasis schon hergeben. Das ist aber sonst genau. Finde ich gut.“	P5
35	“That would be interesting, especially with such inflammatory changes, if inflammation values are stored. This is something I give back to the patients and they tell me and	„Das wäre interessant, gerade bei so entzündlichen Veränderungen, wenn Entzündungswerte hinterlegt sind. Das ist etwas was ich an die Patienten zurückgebe und sagen und	P6

Quotation number	Quotation (translated into English)	Quotation (original German)	Participant Number
	write it down chronologically, for example how the CRP has developed. Because that is hard work. That is of course interesting, if you have a good view of it.”	schreiben sie mir das bitte mal chronologisch auf, wie sich der CRP zum Beispiel entwickelt hat. Weil das ist Fleißarbeit sozusagen. Das ist natürlich, wenn man das schön anschaulich hat, interessant.“	
36	“History is always very helpful. Especially in rare diseases. Because then only the history of the disease provides the information. If you think it's from my field, neurology, psychiatry, for example. With atypical Parkinson's syndromes. If you start with a cross-sectional approach, then you can hardly tell the difference at the bottom of the scale. But then the progression of certain symptoms, like the increase or decrease, especially the increase. This will basically give you the information about the diagnosis. As I mentioned earlier, this varies greatly from illness to illness.”	„Historien sind halt immer sehr hilfreich. Gerade bei Seltenen Erkrankungen. Weil dann nur der Verlauf dann auch die Auskunft gibt. Wenn Sie es jetzt beispielsweise aus meinem Feld, Neurologie, Psychiatrie denken. Bei atypischen Parkinson-Syndromen. Wenn Sie es querschnittlich am Anfang machen, dann können Sie es am Grunde kaum unterscheiden. Aber dann der Verlauf bestimmter Symptome, wie die zunehmen oder das abnehmen vor allem das zunehmen. Geben Ihnen dann eben im Prinzip die Auskunft über die Diagnose. Das ist ja, wie eben schon genannt von Krankheit zu Krankheit sehr unterschiedlich.“	P8
37	“So the parameter should be chosen in such a what, that it really helps me to answer the question.”	„Also der Verlaufsparameter müsste so geschickt gewählt sein, dass er mir wirklich hilft die Frage zu beantworten.“	P2
38	“I think something that is a common symptom of an extremely common disease, you don't need it here. Instead, these should be things that are specifically rare and therefore more specific.”	„Blutdruck ist ja so ein häufiges Symptom. Ich denke so etwas, was ein häufiges Symptom einer außerordentlich häufigen Erkrankung sind, die braucht’s nicht hier. Sondern, das sollten schon Sachen sein, die spezifisch Seltene und damit spezifischer sind.“	P3

Quotation number	Quotation (translated into English)	Quotation (original German)	Participant Number
39	"Yes, an expert should publish on high quality level for this disease. I think that would be important."	„Ja, das er auf hohem Qualitätsniveau für diese Erkrankung publiziert hat. Ich glaube das wäre wichtig."	P3
40	"Well, I would ask further questions. Because the SE Atlas hasn't helped me that much personally as a finding tool. But the direct contact with other colleagues who then support me there. Which I usually confirm with a Medline search, because my thoughts are that someone who knows a lot about a rare disease has published about it."	„Also ich würde mich da schon weiter erkundigen. Weil der SE-Atlas mir persönlich als Findungstool nicht so viel geholfen hat. Sondern der direkte Kontakt mit anderen Kollegen, die mich dann da unterstützen. Die ich dann meistens da mit einer Medline Suche bestätige, weil da meine Gedanken sind, dass jemand, der sich mit einer Seltenen Erkrankung gut auskennt, dazu auch publiziert hat."	P2
41	"Show similar patients, that's okay."	„Ähnliche Patienten anzeigen, das ist okay."	P1
42	"It's all very clear to me."	„Das ist alles sehr gut ersichtlich finde ich."	P7
43	"Now I see, I think it's good, such a timeline Okay, with the timeline. I think timelines in general are useful. You can also see when something happened and in what quantity. You can see that relatively quickly. Exactly. This is now the patient history of the patient. And symptoms. Right here I have coughing, tachypnea (...). Right (…). Ah, you can pick and choose individual symptoms."	„Jetzt sehe ich, finde ich gut, so eine Timeline, die ich hier sehe. Okay, mit der Timeline. Ich finde Timelines generell sind sinnvoll. Man sieht ja auch wann was war und in welcher Menge auch. Das sieht man relativ schnell. Genau. Das ist jetzt quasi die Patientenhistorie der Patientin. Und Symptome. Genau hier habe ich Husten, Tachypnoe (…). Genau (…). Ah, man kann einzelne Symptome sozusagen auswählen."	P5
44	"That's interesting. But like I said, it's all relative."	„Das ist schon interessant. Aber wie gesagt, das ist alles relativ."	P4
45	"And then the time line is all right. You can also see the date of birth	„Und dann ist der Zeitstrang schon in Ordnung. Man sieht ja auch hier das	P7

Quotation number	Quotation (translated into English)	Quotation (original German)	Participant Number
	here. Otherwise, I wondered why this would start. But I see the date of birth here and nothing happens before that. That's why.”	Geburtsdatum. Sonst hätte ich mich jetzt gewundert, warum das hier sozusagen anfängt. Aber ich sehe ja hier das Geburtsdatum und davor passiert nichts. Von daher.“	
46	“And here is also a timeline. Okay. Interesting, yes. Very nice.”	„Und hier dann auch dieser Zeitstrahl. Okay. Spannend, ja. Sehr schön.“	P8
47	“It be great to include several patients in the selection. Not only the most similar, but also for both views.”	„Toll wäre natürlich auch die Mehrzahl mehrerer Patienten sozusagen in die Auswahl mit einzubeziehen. Nicht nur den ähnlichsten, sondern auch für beide Fenster.	P5
48	“Now I am here in the view where the two patients are actually mapped next to each other. With our wife Fiona Bergmann, that makes sense.”	„Jetzt bin ich hier in der Darstellung wo eigentlich die beiden Patienten nebeneinander gemappt sind. Bei unserer Frau Fiona Bergmann, das macht ja Sinn.“	P5
49	“Then the family history again, age at diagnosis, okay the patient. I don't know, it would make sense to repeat the patient's basic demographic data here. Okay, these are below. I'd like to keep them on top.”	„Dann kommt wieder die Familienanamnese, Alter bei Diagnose, okay die Patientin jetzt sozusagen. Ich weiß nicht, hier würde nochmal Sinn machen, dass man die Basis demographischen Daten der Patientin hier nochmal. Okay, die kommen jetzt hier unten. Ich hätte die ganz gerne immer ganz oben.“	P5
50	“I never would have thought of that in a lifetime. I'm thinking more about options for me as a user or something like that. Also the symbol is not (...). So there I would (...).”	„Da wäre ich im Leben nicht draufgekommen. Da denke ich eher an Optionen für mich als Benutzer oder sowas hätte ich da gedacht. Auch das Symbol ist nicht (…). Also da würde ich (…).“	P1
51	“It's not intuitive.”	„Das ist nicht intuitiv.“	P2

Quotation number	Quotation (translated into English)	Quotation (original German)	Participant Number
52	“Well, that's the way it is. I learn it when is use it. But I have to do it regularly, otherwise (...).”	„Gut, bei manchen Sachen ist das halt so. Ich lern das, wenn ich mit irgendetwas umgehe. Aber ich muss es dafür regelmäßig machen, sonst (…).“	P4
53	“Yes, of course, that's always the case, once you know how it works, then it works. But if you haven't done it yet, it's not quite self-explanatory.”	„Ja, das ist natürlich immer so, wenn man einmal weiß wie es geht, dann geht es. Aber wenn man es noch nicht gemacht hat, dann ist es nicht ganz selbsterklärend.“	P6
54	“Right. That's what I mean. This could be shown in a table at the beginning (...).”	„Genau. Das meine ich. Das könnte man schon in einer Tabelle am Anfang darstellen (…).“	P5
55	“Show results (...). I think. Here, directly after the level, I should be able to filter for what I want to filter (...).Not show similar patients. But rather what do I actually want to filter for (...). So here in another colour, another bar above it (...).”	„Ergebnisse anzeigen (…). Ich finde. Hier müsste jetzt direkt sozusagen nach der Ebene sofort oben kommen nach was ich filtern möchte (…]. Also nicht jetzt, ähnliche Patienten anzeigen. Sondern nach was will ich eigentlich filtern (…). Also hier sozusagen in einer anderen Farbe noch ein Balken drüber (…).“	P7
56	“No, not very intuitive. That what I did earlier. Oh, that was not related to the patient at all. Then it's perfectly fine. Then it is. You only have to know it once and then. Perfect. Okay.”	„Nein, nicht sehr intuitiv. Dass was ich vorhin gemacht habe. Ach, das war gar nicht auf den Patienten bezogen. Dann ist es ja völlig gut. Dann ist es ja so. Man muss es nur einmal wissen und dann. Perfekt. Okay.“	P7
57	“What is a scatterplot? Something with dots (...). What do I do with a scatterplot? (…). What I would at least expect to get something there. Then I can click on something, the dot turns red and I don't see anything at all (...). That means here I click 5 times and then I leave it. So if	„Was ist ein Scatterplot? Irgendetwas mit Punkten (…). Was mache ich mit einem Scatterplot? (…). Was ich zumindest erwarten würde, irgendwas dort zu kriegen. Dann kann ich auf irgendetwas klicken, der Punkt wird dann Rot und ich sehe erstmal gar nichts (…). Das heißt	P1

Quotation number	Quotation (translated into English)	Quotation (original German)	Participant Number
	you click very closely, you can see that something changes up there, but somehow. Any information about the patient, diagnosis or something. Otherwise it's not intuitive."	hier klicke ich 5 Mal und dann verlasse ich das wieder. Also wenn man ganz genau hin klickt, sieht man, das sich da oben irgendetwas verändert, aber irgendwie. Irgendwelche Informationen zu dem Patienten, Diagnose oder irgend sowas. Sonst finde ich das gar nicht intuitiv."	
58	"You now expect the same data only in a different display format. The axes here should be labelled. The X-axis with the degree of the match. Here on the Y-axis we have no axis labeling at all. Ah, and then, when I click on the individual points of the scatterplots, the other patients will be shown (...)."	„Man erwartet jetzt dieselben Daten nur in einer anderen Anzeigeform. Genau, jetzt fällt mir hier auf. Dass die Achsen hier sollten beschriftet sein. Also zumindest jetzt hier die. Die X-Achse mit dem Grad der Übereinstimmung. Hier auf der Y-Achse haben wir gar keine Achsenbeschriftung. Ah und dann finde ich sozusagen bei Klick auf die einzelnen Punkte des Scatterplots, werden mir sozusagen die anderen Patienten angezeigt (…)."	P5
59	"The question is now, if the point could be clicked here. Then now is not bad. So let's put it this way. Of course it is quicker to find out who was ranked. But actually, to be honest, the table, because then you can see the information all at once. The other one is nice. I think that becomes interesting when, or would be interesting if there were more patients. As if there are now somehow 10. When there are 100, for example. So, if the similarity values are close together. Then, of course, a scatterplot gives me the additional information."	„Die Frage ist ja jetzt, wenn jetzt hier der Punkt anklickbar wäre. Dann ist es jetzt nicht schlecht. Also sagen wir mal so. Man erfasst natürlich schneller wer geranked ist. eigentlich, also ehrlich gesagt die Tabelle, weil man dann auf einmal die Informationen sieht. Das andere ist nett. Ich glaube das wird dann interessant wenn, wenn es dann tatsächlich, oder wäre dann interessant, wenn es noch mehr wären. Als, wenn es jetzt irgendwie 10 sind. Wenn es z.B. 100 sind. Also, wenn die Ähnlichkeitswerte nah beieinanderliegen. Dann	P8

Quotation number	Quotation (translated into English)	Quotation (original German)	Participant Number
		bringt mir ein Scatterplot natürlich die Zusatzinformation.“	
60	“That means I have to rely on an unknown algorithm and then have to find out for myself in the symptoms, how the algorithm calculated this.”	„Das heißt ich muss mich auf einen unbekannten Algorithmus verlassen und muss dann selber in den Symptomen herausfinden, warum er das jetzt ausgerechnet hat.“	P1
61	“Now the user of course asks himself, I would ask myself the question, we now have a similarity of 74.8 %. So, the value is not really transparent for me or now as a medical user, how this happens. How is the similarity calculated.”	„Jetzt stellt sich der User natürlich, ich würde mir jetzt die Frage stellen, wir haben jetzt hier eine Ähnlichkeit von 74,8 %. Also der Wert ist für mich oder jetzt als ärztlicher Benutzer nicht wirklich transparent, wie es dazu kommt. Auf welcher Basis ist diese Ähnlichkeit.“	P5
62	“And now my question. There have been a number of diagnoses (...). What is being compared, the diagnosis or the symptoms?”	„Und jetzt meine Frage. Es hat diese Diagnosen reihen gegeben (…). Was wird denn verglichen, die Diagnose oder die Symptome?“	P3
63	“Similarity of phenotypes. I would expect an explanation now. At least a click that I can have displayed, what did he compare. And why are these diagnoses 49.2 % similar. Some idea. Otherwise this is a real black box.”	„Ähnlichkeit der Phänotypen. Da würde ich jetzt eine Erklärung erwarten. Zumindest ein Klick, dass ich mir anzeigen lassen kann, was hat er denn da verglichen. Und warum sind diese Diagnosen zu 49,2 % ähnlich. Irgendeine Idee, die ich habe. Sonst ist das eine echte Blackbox.“	P2
64	"Where do I find this diabetic patient now, with the comparison? There is diabetes mellitus type 2, a very common disease in older people. So where do I find this similarity with these symptoms? Which are present in diabetes and which are not?”	„Wo finde ich jetzt diesen Diabetiker, mit dem Vergleich? Aber jetzt wo sehe ich dann. Es gibt ja Diabetes Mellitus Typ 2. Eine sehr häufige Erkrankung bei älteren Personen. Wo finde ich jetzt dann, wo die Ähnlich-	P3

Quotation number	Quotation (translated into English)	Quotation (original German)	Participant Number
		keit ist mit diesen Symptomen? Welche sind bei Diabetes vorhanden und welche sind nicht?“	
65	“In the case of diagnoses, it would be interesting to see which diagnoses match. So because if there is any allergy, allergic asthma is not as interesting as any other diagnosis. That would be quite good, if what is matched is still differentiated.”	„Bei den Diagnosen wäre natürlich interessant, welche Diagnosen da übereinstimmen. Also, weil wenn es irgendeine Allergie ist, das allergische Asthma ist nicht so interessant wie irgendeine andere Diagnose. Das wäre ganz gut, wenn das noch differenziert ist, was da matched.“	P6
66	“I mean, you have done it in such a way that the similarity in percent for the different phenotypes, diagnoses, family history, etc. Can you split this up here, that when I click on it here, it shows me which phenotypes are still similar here (...). In any case. Because afterwards I think to myself, oh this is a super similar patient and in the end these are exactly the diagnoses that match, that is not of interest.”	„Ich meine du hast das jetzt hier quasi so gemacht, das die Ähnlichkeit in Prozent für die verschiedenen sozusagen, Phänotypen, Diagnosen, Familienhistorie usw. Kannst du das dann hier noch aufsplitten, das wenn ich dann hier noch drauf klicke, dass er mir anzeigt, welche Phänotypen dann hier noch ähnlich sind (…). Auf jeden Fall. Weil hinterher denke ich mir, oh das ist eine super ähnliche Patientin und letztendlich sind das genau die Diagnosen, die da übereinstimmen, die mich nicht interessieren.“	P7
67	“The displayed parameters. The anonymous patient was 63 years old when she was diagnosed. Similarity between the patients. Exactly here I miss again, which diagnosis have exactly matched?”	„Die dargestellten Parameter. Die anonyme Patientin war 63. Vor einem Jahr gestellt. Ähnlichkeit zwischen den Patienten. Genau hier fehlt mir wieder sozusagen. Welche Diagnosen genau gematcht haben?“	P5
68	“This is basically a good feature. If I have not done a similarity analysis now, then I cannot get an expert with	„Das ist grundsätzlich ein gutes Feature. Wenn ich jetzt keine Ähnlichkeitsanalyse gemacht habe, dann kann ich aber auch nicht nach einem	P1

Quotation number	Quotation (translated into English)	Quotation (original German)	Participant Number
	a suspicious diagnosis (...). That would be another nice feature.”	Experten suchen mit einer Verdachtsdiagnose (…). Das wäre noch ein schönes Feature.“	
69	“Well, in my opinion, the link would be more helpful if you have some possibilities to enter differential diagnoses. And with this diagnosis, you can then search for the experts. But you also want to search for self-considered diagnoses.”	„Also, da wäre eher aus meiner Sicht die Verknüpfung hilfreich, wenn man selber irgendwelche Möglichkeiten hat Differenzialdiagnosen zu hinterlegen. Und anhand dieser Diagnose man dann die Experten suchen kann. Aber man will ja auch mal so für selbstüberlegte Diagnosen suchen.“	P6
70	“I would to see a Spider-Chart. Where you can show the individual components as dimensions. Symptoms, diagnosis, family.”	„Ich hätte ja am liebsten, also was ich mir gewünscht hätte, wäre so ein Spider-Chart sozusagen. Wo sie als Dimensionen sozusagen die einzelnen Komponenten auftragen, was weiß ich. Symptome, Diagnosen, Familie.“	P5
